# Phase Behavior of Light-Responsive Lyotropic Liquid Crystals for Molecular Solar Thermal Energy Storage

**DOI:** 10.1021/jacs.5c09267

**Published:** 2025-08-04

**Authors:** Beatrice E. Jones, Zhihang Wang, Martijn A. Zwijnenburg, Charlotte J. C. Edwards-Gayle, Kasper Moth-Poulsen, Nathan Cowieson, Rachel C. Evans

**Affiliations:** † Department of Materials Science & Metallurgy, 2152 University of Cambridge, 27 Charles Babbage Road, Cambridge CB3 0FS, United Kingdom; ‡ Diamond Light Source, Harwell Science and Innovation Campus, Didcot OX11 0DE, Oxfordshire, United Kingdom; § Department of Chemistry, 4919 University College London, 20 Gordon Street, London WC1H 0AJ, United Kingdom; ∥ The Institute of Materials Science of Barcelona, ICMAB-CSIC, Bellaterra, Barcelona 08193, Spain; ⊥ Department of Chemical Engineering, Universitat Politècnica de Catalunya, EEBE, Eduard Maristany 10−14, Barcelona 08019, Spain; # Catalan Institution for Research & Advanced Studies, ICREA, Pg. Lluís Companys 23, Barcelona 08010, Spain

## Abstract

Molecular solar thermal energy storage (MOST) materials are a promising method for renewable energy storage that captures solar energy and releases it on demand as heat. Azobenzene is attractive for MOST applications due to its photoreversible *E–Z* isomerization. Recently, phase-change materials have been formed using azobenzene to increase their energy-storage capacity; however, these condensed phases often lower the isomerization degree, which is only recovered on dissolution. In this work, sparing solvent addition is used to drive the self-assembly of azobenzene photosurfactants (AzoPS) into lyotropic liquid crystal (LLC) phases, which are explored for MOST applications for the first time. Using small-angle X-ray scattering (SAXS), polarized optical microscopy, and differential scanning calorimetry (DSC), we show that the structure-isomerization behavior, and energy-storage properties of these light-responsive LLCs can be systematically tuned by adjusting the photosurfactant structure, solvent, and concentration. Furthermore, by developing a method that combines SAXS with *in situ* DSC, we directly correlate the isomerization-induced LLC phase transitions to their energy-storage contributions. The formation of LLC phases through solvent addition both enhances the degree of isomerization (by up to 20%) and amplifies the structural disordering on isomerization, resulting in energy-storage densities of up to 123 J g^–1^. The ability to tune both the structure and isomerization properties in LLC materials suggests significant promise for MOST applications. In addition, the combination of advanced characterization methods used to establish the structure–isomerization–enthalpy (LLC-photoswitch-phase change) relationships provides unique insight into these multicomponent systems and accelerates the design pathways to future iterations for competitive solar energy storage devices.

## Graphical abstract

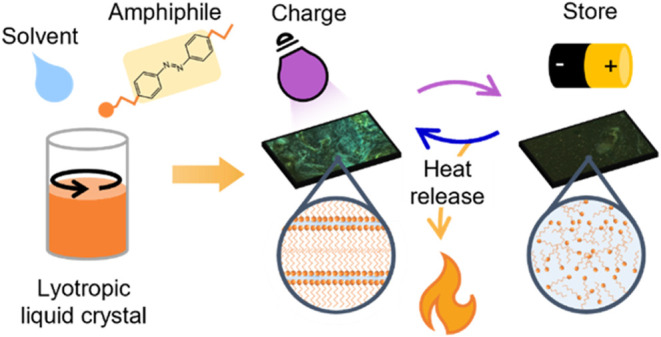

## Introduction

There is a critical need to decrease our dependence on fossil fuels in response to global warming. As the most abundant energy resource on Earth, the Sun provides more than enough energy to meet the global demand;^1^ however, to overcome its temporal, seasonal, and meteorological dependence, storage methods are needed to allow energy supply on demand. Molecular solar thermal energy storage (MOST) offers a promising method to capture and store solar energy using photoswitchable materials.^2–4^ In MOS systems, energy is captured through chemical photoisomerization into a higher-energy state, where it can be stored and later released on-demand as heat, using a thermal or catalytic trigger.^2^ Many photoswitchable molecules have been investigated for MOST, including norbornadiene,^5,6^ dihydroazulene vinylheptafulvene,^7,8^ fulvalene-diruthenium complexes,^9,10^ and azo­(hetero)­arenes.^11,12^ Of these, azobenzene and its derivatives have received particular attention due to their highly cyclable, *E* (*trans*)-*Z* (*cis*) isomerization, chemical stability, and ease of functionalization.^11,13^ However, in its native form, the energy-storage capacity of azobenzene is limited to its isomerization enthalpy (41 kJ mol^–1^, 225 J g^−1^),^14^ which falls behind the target for MOST materials (368 J g^–1^) to match sodium ion batteries.^3^ To increase the energy stored in azobenzene, various methods have been employed, such as introduction into nanocarbon templates,^15^ polymers,^16,17^ or macrocyclic structures.^18,19^ Alternatively, systems that display a phase transition (*e.g*., solid-to-liquid^20–24^ or solid–solid^25^) on isomerization can increase the energy released when reverse isomerization is triggered.^26^ In this case, the enthalpy of fusion for the ordered phase is added to the stored isomerization enthalpy to give a greater total energy-storage capacity, increasing it by up to 210 J g^–1^ (∼70 kJ mol^–1^).^21^ Despite this, incorporation of the photoswitch into condensed phases can suppress isomerization due to steric hindrance and high optical absorbance,^2,27^ thereby lowering the charging efficiency into the higher-energy state. This is often overcome by dissolving the MOST compound in a suitable solvent before isomerization, but this results in dilution of the photoswitchable energy-storing material and a lower volumetric energy-storage density.^28^ Hence, creating MOST systems with increased energy-storage capacity while retaining a high isomerization efficiency remains a challenge in the field.

Recently, thermotropic liquid crystals (LCs) have garnered interest for MOST applications due to their ability to self-organize into ordered nanostructures for increased energy storage, while retaining a fluid state to aid isomerization.^29–33^ Zhang et al. used cationic azobenzene surfactants with a linear alkyl chain tail and a charged quaternary ammonium headgroup, containing two methyl groups and one of two different bulky ring groups (either phenyl or cyclohexane) attached to the nitrogen.^32^ The surfactants formed crystalline, smectic LC mesophases that displayed phase transitions resulting in increased storage enthalpy. However, despite the addition of bulky head groups to increase the free volume, the low isomerization degree in the solid state (<45%) ultimately limited the storage energy to 131 J g^–1^ (cf. 161 J g^–1^ in solution). Additionally, Gupta and coauthors have studied numerous LC mesophases for MOST applications, including chiral nematic,^34^ discotic nematic,^35^ and columnar,^30,31^ by combining azobenzene moieties with various functionalization into LC-forming mesogens. A storage enthalpy of up to 125 J g^–1^ in a cholesteric liquid crystal was achieved and, notably, the fluidity of the LC phase also aided isomerization in thin films (65 μm), with up to 70% *Z* isomer achievable using filtered sunlight.^34^

In addition to thermotropic mesophases, LCs can also be formed through the addition of solvent, which drives the self-assembly of amphiphile bilayers into structures with long-range orientational order, namely, lyotropic liquid crystals (LLCs).^36^ Previously, a diverse range of LLC mesophases have been formed using azobenzene photosurfactants (AzoPS) with either charged^37^ or neutral head groups,^38–43^ including lamellar, hexagonal, or cubic architectures. In these systems, isomerization of the AzoPS leads to a change in shape and polarity^44^ of the photoswitch, which modifies amphiphile geometry and hydophilicity.^45^ This has a knock-on effect on the AzoPS packing and assembly,^45,46^ and in LLCs can result in a change to the phase dimensions,^42,43^ symmetry,^47^ or destruction of the ordered networks.^37,41,42,48^ Despite this demonstration of light-responsive structural control, little is known about the relationship between structure, isomerization, and enthalpy storage in these systems, meaning LLC mesophases have not been explored for solar-energy storage applications until now.

Herein, we unpick the structure–isomerization–energy storage relationships in light-responsive LLCs and present them as a class of materials for MOST for the first time. The controlled addition of solvent (water or ethylene glycol) increases the isomerization degree in the otherwise dense, thermotropic LC phase, while also aiding self-assembly into ordered LLC phases for greater energy storage due to intermolecular interactions. We investigate three different AzoPS, each containing a neutral tetraethylene glycol headgroup, butyl spacer, azobenzene core, and alkyl tail of increasing length (*n* = 6, 8, or 10), subsequently referred to as C_6_AzoC_4_E_4_, C_8_AzoC_4_E_4_, and C_10_AzoC_4_E_4_ (Figure 1a). Both C_6_AzoC_4_E_4_ and C_8_AzoC_4_E_4_ have been shown to form LLC phases that disorder under ultraviolet (UV) irradiation using polarized optical microscopy (POM),^41^ however, structural analysis of how this disordering occurs, and how it affects the energy storage has never been investigated. To gain an in-depth understanding, we use small-angle X-ray scattering (SAXS), POM, and differential scanning calorimetry (DSC) to show that these AzoPS form thermotropic and lyotropic LCs with tunable properties that depend on the alkyl tail length, solvent, and concentration. Additionally, by combining SAXS with *in situ* DSC, we correlate the structural and enthalpy-storage properties of these materials for the first time. We demonstrate that the introduction of solvent to form lyotropic LCs leads to additional control over the *E–Z* isomerization of molecular AzoPS, which can magnify the LLC order-to-disorder transition while retaining energy-storage densities (up to 123 J g^–1^).

**Figure 1:**
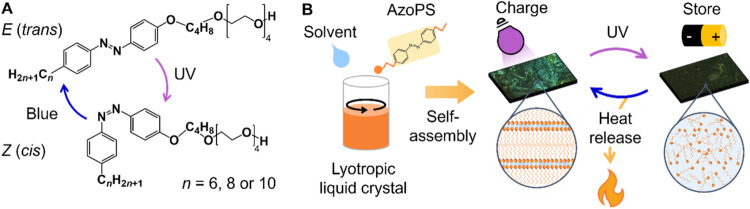
Creation of azobenzene lyotropic liquid crystals. (a) The chemical structure of AzoPS used, showing isomerization with UV (365 nm) and blue (455 nm) light. The alkyl chain length, n, was varied to give three different structures: C_6_AzoC_4_E_4_, C_8_AzoC_4_E_4_, and C_10_AzoC_4_E_4_. (b) Schematic diagram showing the formation of the LLC, internal structure in the native, *E* isomer, and effect of irradiation with UV light, triggering an order-to-disorder transition, which enhances the energy released on reverse isomerization.

## Results and Discussion

### Self-Assembly of AzoPS into Thermotropic Liquid Crystals

The structural and energy-storage properties of the three AzoPS were first investigated for the native, *E* isomer in the solid state (*i.e*., no solvent), as a reference from which to compare the effect of solvent addition and lyotropic LC formation. SAXS measurements revealed that all three AzoPS form smectic LC phases at room temperature (21 °C), shown by the formation of Bragg peaks with *Q* positions in a ratio of 1:2:3 (Figure 2a and Supporting Information, S1). This is supported by a characteristic birefringent, striped pattern in the POM micrographs, due to the molecular anisotropy (Figures 2b and S2), and is consistent with previous observations for AzoPS.^41^ The smectic mesophase is formed from bilayers of AzoPS stacked on top of each other in a lamellar arrangement. The surfactant tails may be packed rigidly or fluidly, giving rise to crystalline (*L*_c_) or fluid (*L*_α_) lamellar arrangements (Figure 2c). Here, the interlamellar spacing (*d*) closely matches twice the AzoPS tail length (see SI, Table S2 and Section S4), providing a method to tune the nanostructure using the alkyl chain length. On heating, all AzoPS undergo a smectic-to-isotropic (*I*_0_) transition at a clearing temperature (*T*_i_) between 56 and 90 °C. This is shown by the loss of SAXS peaks and birefringence (Figure 2, and SI, Section S4) and supports the temperature-dependent thermotropic LC assignment.

**Figure 2:**
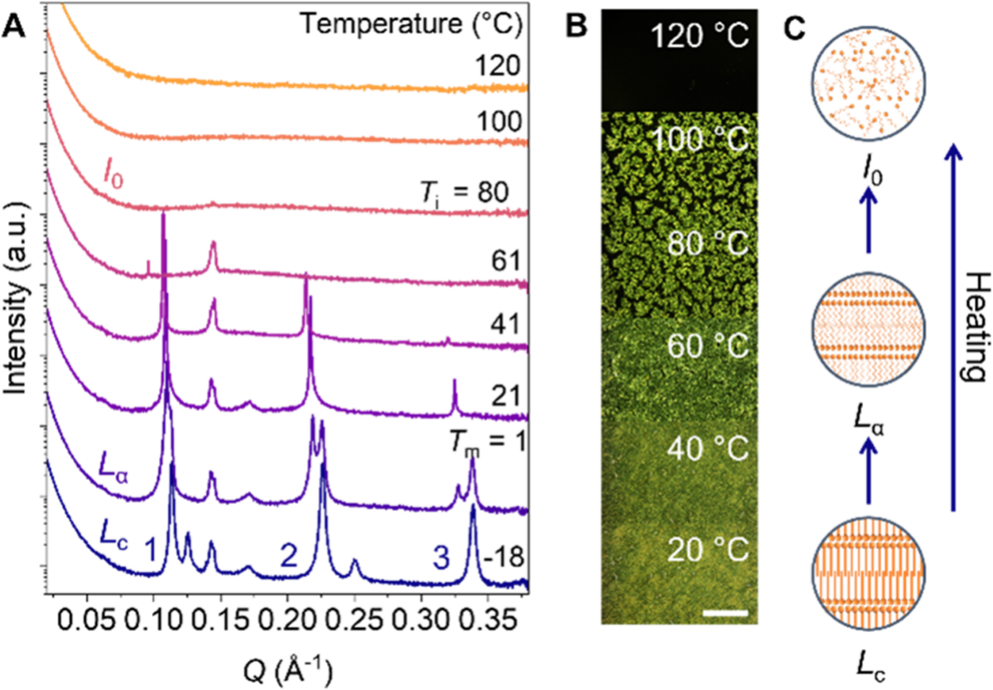
Formation of thermotropic LCs by using AzoPS. (a) SAXS patterns for C_8_AzoC_4_E_4_ on heating from −18 to 120 °C showing the formation of ordered, smectic phases, which undergo a crystalline-to-fluid (*L*_c_–*L*_α_) transition at the melting temperature (*T*_m_) and a smectic-to-isotropic (*I*_0_) transition at the clearing temperature (*T*_i_). (b) POM micrographs also show the *L*_α_–*I*_0_ transition on heating as a loss of the bright, birefringent pattern. The scale bar indicates 1 mm. (c) Schematic diagrams showing the AzoPS packing in the *L*_c_, *L*_α_, and *I*_0_ phases.

The additional energy storage available for MOST will be governed by the phase-change enthalpies due to disordering and reordering of the LC phases. Phase transitions were investigated across a temperature range of −20–150 °C using SAXS with *in situ* pseudo-DSC to allow direct comparison between phase transitions and the associated enthalpy changes. We note that to quantify these enthalpy changes, *ex situ* DSC scans were also performed on identical samples, as the *in situ* DSC setup does not have a reference pan. For the intermediate chain length, C_8_AzoC_4_E_4_, a peak shift in the SAXS shows that a phase transition occurs at 0 °C (Figure 2a). This is associated with an increase in *d*, suggesting a *L*_c_–*L*_α_ transition that results in fluidization of the surfactant tails within the amphiphile bilayer plane, increasing the spacing to accommodate this increased movement (Figure 2c and SI, Section S4).^49^ This phase change, at the melting transition temperature (*T*_m_), is associated with endothermic peaks in both *in-* and *ex situ* DSC thermograms (Figures 3 and S3), with an enthalpy change of 22 J g^–1^ (SI, Table S6). On heating to 30 °C, a further endothermic enthalpy change of 11 J g^–1^ is observed, which can be assigned to the *L*_α_–*I*_0_ transition (SI, Table S6). The *L*_α_ and *L*_c_ smectic phases can be reformed on cooling, which is accompanied by exothermic peaks in the DSC, at the fluid (*T*_f_) and crystalline (*T*_c_) transition temperatures (Figure 3). A second heating cycle shows that the *L*_c_–*L*_α_ and *L*_α_–*I*_0_ transitions are reproducible over multiple cycles with comparable enthalpy changes (SI, Figure S4).

**Figure 3:**
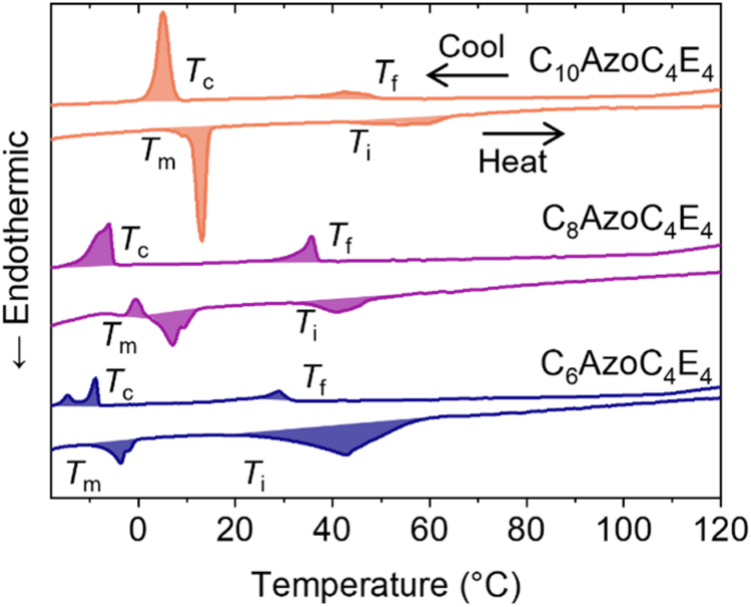
DSC thermograms for AzoPS show endothermic peaks on heating that can be assigned to crystalline-fluid lamellar (*T*_m_) and lamellar–isotropic (*T*_i_) phase transitions and exothermic peaks on cooling that can be assigned to isotropic-fluid lamellar (*T*_f_) and fluid-crystalline lamellar (*T*_c_) transitions.

AzoPS of both shorter and longer tail lengths (*i.e*., C_6_AzoC_4_E_4_ and C_10_AzoC_4_E_4_) were similarly investigated and display comparable *L*_c_–*L*_α_ and *L*_α_–*I*_0_ transitions (see SI, Section S4). Both *T*_m_ and *T*_i_ increase with alkyl tail length, with *T*_i_ increasing by roughly 10 °C with every two carbons added to the tail (Figure 3). This can be attributed to an increase in the intermolecular interactions between the AzoPS tails with increasing chain length, resulting in a greater thermal barrier to tail fluidization (at *T*_m_) or disordering (*T*_i_). Furthermore, the *L*_c_–*L*_α_ phase-change enthalpy also increases by ∼10 J g^–1^ for every two carbons added to the alkyl chain (see the SI, Table S6). Interestingly, the alkyl chain length has little effect on the enthalpy change due to the *L*_α_–*I*_0_ transition, which remains at around 10 J g^–1^. This can be rationalized as the *L*_c_–*L*_α_ phase transition is dominated by the fluidization of the AzoPS tail groups, meaning that the intermolecular interactions between the alkyl tails will directly affect this transition enthalpy. Once in the fluid lamellar phase, the *L*_α_–*I*_0_ transition is much more influenced by the separation of the head groups, which we have not modified in this study.

### Effect of Solvent Addition to form Lyotropic Liquid Crystals

Having determined that AzoPS forms thermotropic LCs, we next investigated the addition of solvent to drive self-assembly into lyotropic LCs. Initially, water was used as the solvent since water-AzoPS LLCs have been reported previously.^41^ The concentration of AzoPS in water was varied from 10 to 90 wt %, in 20 wt % increments. For 10–30 wt % C_8_AzoC_4_E_4_, SAXS patterns show broad interaction peaks, characteristic of an isotropic micellar phase (*I*_0_) with strong interparticle interactions, consistent with previous reports (Figure 4a).^41^ This is supported by the black POM micrograph with a few bright, birefringent spots, from ordered crystallites (Figure 4b). On increasing to 50 wt %, AzoPS self-assembles into a lamellar mesophase (*L*_α_), as evidenced by the formation of peaks at a *Q* ratio of 1:2 in the SAXS and a bright, smoke-like pattern in POM (Figure 4). Here, the large *d* spacing (10.3 nm, see SI, Table S7) suggests a swollen lamellar phase (*L*_α,s_) with wide water channels between the AzoPS sheets. At 70 and 90 wt %, the Bragg peaks become much sharper, with a *Q* ratio of 1:2:3, showing increased lamellar ordering (Figure 4a), visible as a densely packed, birefringent POM image (Figure 4b). The *Q* positions of the Bragg peaks shift to higher values with increasing AzoPS concentration, indicating a decrease in the *d* spacing of the lamellae (SI, Table S7), which could be due to a dehydration of the head groups within the phase.^50^

**Figure 4:**
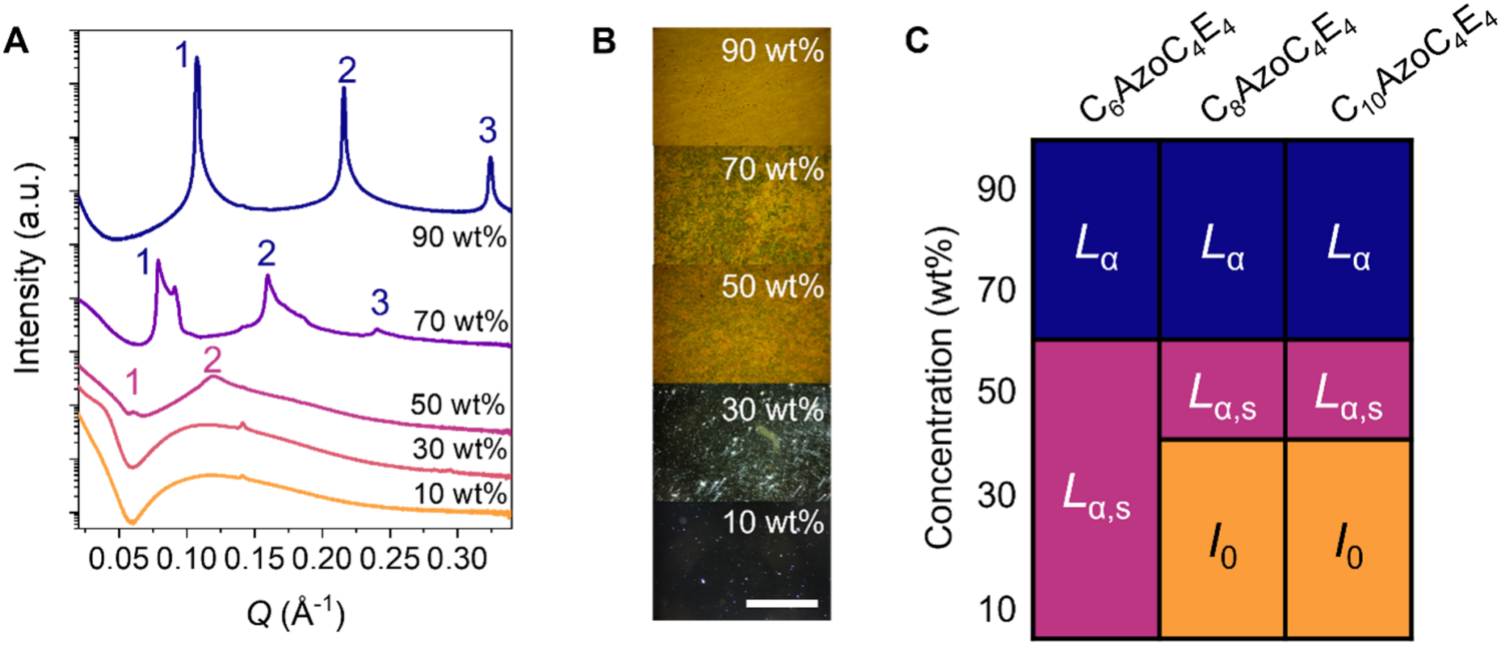
Formation of LLCs on increasing concentration of AzoPS in water as shown for C_8_AzoC_4_E_4_ by the formation of (a) Bragg peaks in the SAXS pattern for 50–90 wt % and (b) birefringent smoke-like patterns under POM. The scale bar indicates 1 mm. (c) Partial phase diagram for each of the AzoPS showing the presence of isotropic (*I*_0_), swollen lamellar (*L*_α,s_), and lamellar (*L*_α_) mesophases on increasing concentration.

AzoPS of shorter and longer tail lengths (*i.e*., C_6_AzoC_4_E_4_ and C_10_AzoC_4_E_4_) also form LLC mesophases (Figure 4c, for full discussion see SI, Section S5). The alkyl chain length has an important effect on the concentration at which self-assembly into lamellar mesophases is onset, with higher concentrations (>50 wt %) required for longer alkyl chains. As seen for the thermotropic LCs, the interlamellar spacing increases with alkyl chain length (SI, Table S7). Nevertheless, all AzoPS follow similar trends in forming swollen lamellar phases (*L*_α,s_) at lower concentrations, which become denser, *L*_α_ phases on increasing concentration.

As the intermediate chain length in this study, C_8_AzoC_4_E_4_ was chosen for *in situ* DSC analysis with SAXS to match phase transitions to enthalpy changes in LLCs at 50, 70, and 90 wt % in water. For 50 wt %, no LLC ordering was observed (SI, Figure S12), highlighting the sensitivity of this concentration to sample loading, as it is at the *I*_0_–*L*_α_ phase boundary. At 70 wt %, SAXS shows that C_8_AzoC_4_E_4_ forms lamellar mesophases across the temperature range −20 to 100 °C (Figure 5a). However, numerous phase transitions are present, as visible from the growth and simultaneous loss of sets of peaks, while the characteristic lamellar ratio is always retained. At 0 °C, a transition to a phase with a smaller *d* spacing suggests a *L*_c_–*L*_α_ phase transition (Figure 5 and SI, Table S8),^49^ analogous to that observed for the thermotropic system. A small, endothermic peak in the *in situ* DSC can be assigned to this lamellar reordering (*T*_m_, Figure 5d); however, the *ex situ* DSC displays both exothermic (−12 J g^–1^) and endothermic (11 and 3 J g^–1^) peaks in this region (Figure 5e and SI, Table S10). This shows that there is an interplay between the ordering of the lamellar phases (exothermic) as well as the melting or fluidization of the AzoPS tail groups (endothermic). At 100 °C, the *L*_α_ phase is lost due to simultaneous melting and evaporation of the water, visible as a loss of birefringence under POM, loss of Bragg peaks in SAXS, and a corresponding endothermic peak in the*in situ* DSC thermogram (Figure 5, *T*_i_). Comparable phase transitions were also observed at 90 wt % in water (see SI, Section S6 for full discussion), with the *L*_α_–*I*_0_ transition associated with an endothermic enthalpy change of 8 J g^–1^ (SI, Table S10). This endothermic enthalpy change on disordering suggests that AzoPS LLCs would be suitable materials for MOST applications, providing additional energy storage through self-assembly into ordered mesophases.

**Figure 5:**
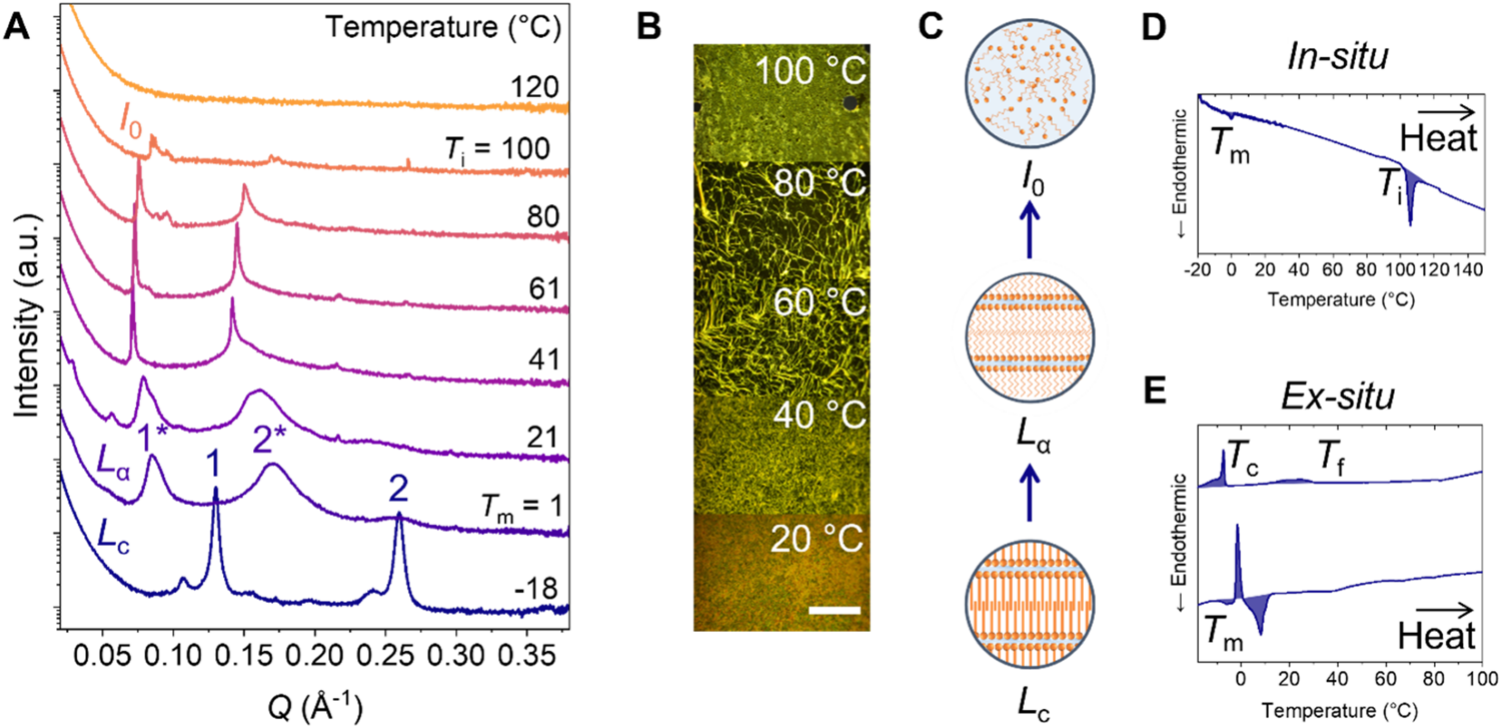
Effect of temperature on LLC mesophases. On heating the 70 wt % C_8_AzoC_4_E_4_ LLC, lamellar–lamellar (*L*_c_–*L*_α_) and lamellar–isotropic (*L*_α_–*I*_0_) transitions can be seen in the (a) SAXS at the temperatures labeled *T*_m_ and *T*_i_, respectively, and (b) POM. The scale bars on the POM micrographs indicate 1 mm. (c) Schematic diagrams showing the morphology of the crystalline (*L*_c_) and fluid (*L*_α_) lamellar, and isotropic (*I*_0_) LLC phases. The phase changes are associated with endothermic enthalpy changes measured using (d) *in situ* and (e) *ex situ* DSC.

While water is the most widely studied solvent for LLC formation, it is not ideal for MOST applications, as its high specific heat capacity would lead to absorption of the heat released from the AzoPS on reverse isomerization. Owing to its very low specific heat capacity (1.69 J g ^–1^ K^–1^),^51^ toluene is the most frequently used solvent for MOST systems; however, there are concerns over its toxicity and flammability.^52^ As an additional test, we further investigated the formation of AzoPS LLCs using ethylene glycol as the solvent, which has a low flammability, a lower specific heat capacity than water (2.38 cf. 4.18 J g^–1^ K^–1^, respectively),^53^ and a higher boiling point (197 cf. 100 °C),^54^ reducing the risk of sample dehydration during use. C_8_AzoC_4_E_4_ and C_10_AzoC_4_E_4_ both formed *L*_α_ LLC phases at concentrations of 50 wt % and above, visible using SAXS and POM (see SI, Section S7). This shows that alternative solvents can be used to create LLCs using AzoPS, expanding the possibilities for materials design, especially for MOST applications.

### Effect of Solvent on Isomerization in AzoPS Liquid Crystals

Having determined that AzoPS self-assemble into both thermotropic and lyotropic LC phases, we next investigated the effect of LC phase formation on isomerization. The degree of isomerization under UV (365 nm) irradiation for each AzoPS was first determined by ^1^H nuclear magnetic resonance (NMR) spectroscopy in solution (DMSO-*d*_6_, 10 mM) to eliminate the effects of steric hindrance. All AzoPS molecules reached a photostationary state (PSS) of >95% *Z* isomers within 30 min of UV irradiation (SI, Figure S17). In addition to this, using UV-visible (UV–vis) absorbance spectroscopy, the photoswitch showed high cycling stability when dissolved in water, over 20 UV and blue cycles (Figure 6a), and over 24 h of constant UV irradiation, with no evidence of decay of the intensity of the absorbance maximum peak (SI, Figure S21).

**Figure 6:**
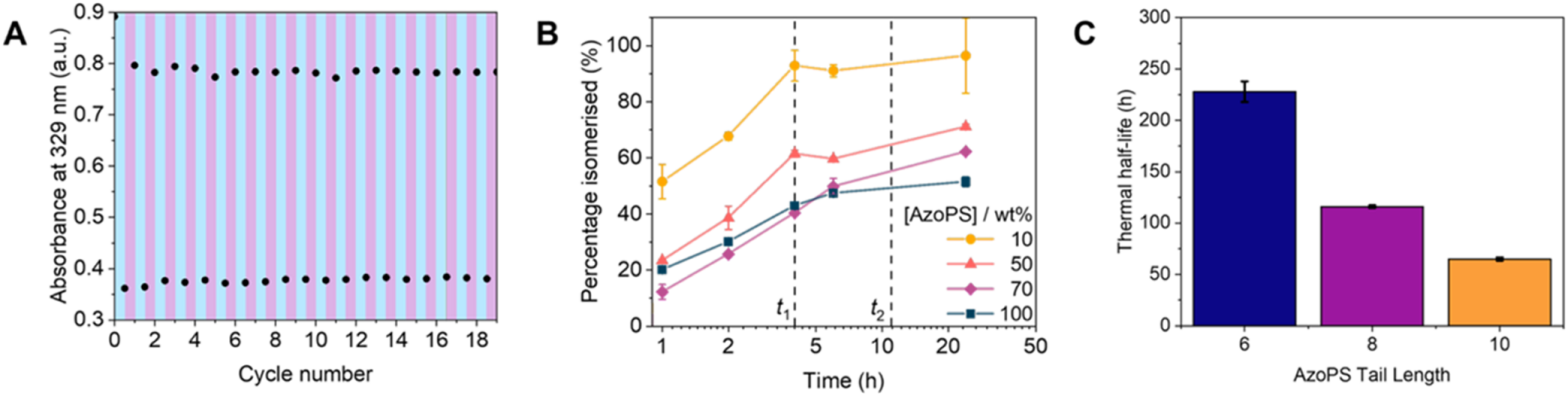
Isomerization behavior of AzoPS. (a) Absorbance at the *E* absorbance maximum (329 nm) against cycle number for C_8_AzoC_4_E_4_ (50 μM in water) on irradiation with UV (365 nm) and blue (455 nm) light to obtain the *E* and *Z*-rich PSS. (b) The percentage isomerized (*E–Z*) after a given UV irradiation time increases with decreasing concentration of C_8_AzoC_4_E_4_ in D_2_O LLCs. Times *t*_1_ and *t*_2_ correspond to irradiation times used for SAXS data collection. (c) The thermal half-life at room temperature (20 °C) decreases with increasing AzoPS tail length (from C_6_AzoC_4_E_4_ to C_10_AzoC_4_E_4_).

To determine the degree of isomerization in the thermotropic LC mesophase, AzoPS were irradiated in the pure state from the top-down in a DSC pan to replicate the irradiation conditions for the other studies. The AzoPS were then dissolved in DMSO-*d*_6_ for NMR measurement. Irradiating in the thermotropic LC state has a significant effect on the isomerization degree, with the AzoPS only reaching a maximum of 52–58% Z isomers after 24 h of irradiation (SI, Figure S17). However, the degree of isomerization does not change significantly (≤10%) beyond 6 h irradiation for all AzoPS. While this is lower than the maximum degree of isomerization for azobenzene LCs in previous studies (70%),^34^ it is worth noting that this was achieved using a thin film (65 μm) of sample, in comparison to the bulk used here, and it is conceivable that a much higher isomerization degree could be achieved on decreasing the sample thickness to reduce inner filter effects. The lower degree of isomerization in the condensed LC phase is a result of both the lower free volume available for isomerization, resulting in steric hindrance,^27^ and the high absorbance and low diffusivity in these viscous phases. This means that only the topmost area of the sample becomes isomerized, due to the high molar absorption coefficients (ε*_E_*_,365nm_ ∼70,000 M^–1^ cm^–1^) for these molecules and therefore low penetration depth of the UV light.

We next investigated whether the addition of water to form LLCs affected the degree of isomerization for AzoPS, specifically using the intermediate chain length C_8_AzoC_4_E_4_. Since the isomerization degree was determined using NMR spectroscopy, D_2_O was used as the solvent, instead of H_2_O. This swap is nontrivial as the change in dipole moment will affect the hydrogen bonding interactions, which are crucial for the self-assembly properties in LLCs.^55^ The effect of this switch in solvent was investigated using SAXS and POM, with comparable LLC mesophases forming at the respective concentrations (for full discussion, see SI, Section S9). Three different concentrations were investigated, 10, 50, and 70 wt %, corresponding to the *I*_0_, *L*_α,s_, and *L*_α_ phase regions. It was found that both the final isomerization degree (after 24 h of UV irradiation) and the rate of isomerization decrease with increasing concentration of AzoPS (Figure 6b). This is significant as, even within the concentration range where the AzoPS are self-assembled into LC states (≥50 wt %), the degree of isomerization can still be controlled, reaching as high as 71% at 50 wt %, which is comparable to the maximum previously reported for thin film samples.^34^ This means that the degree of isomerization can be increased within MOST materials without disrupting the ordered self-assembled phases that are needed to enhance the energy-storage properties.

The thermal half-life of the photoswitch is important for MOST applications, providing an indication of the storage stability of the material once it has been charged into the *Z* isomer. To calculate this, Eyring analysis was performed for each AzoPS by taking time-dependent UV–vis absorbance spectra at temperatures from 45 – 65 °C, as the *Z* isomer relaxes back to the *E* isomer (see SI, Section S10). It was found that the enthalpy change for *Z–E* isomerization, Δ*H*, decreases with an increase in alkyl chain length. This results in a shorter thermal half-life at 20 °C, decreasing from 228 to 65 h when the alkyl chain length increases from C_6_AzoC_4_E_4_ to C_10_AzoC_4_E_4_ (Figure 6c). This means that not only do we have a method to control the isomerization degree within these self-assembled materials, but we can also use the chemical structure to further tune the thermal half-life.

### Effect of Isomerization on Liquid Crystal Structure and Energy Storage

The effect of isomerization on the LC structure was next investigated. This is vital for MOST applications because reversible phase changes that occur on isomerization can be linked to additional stored enthalpy.^21^ To probe this, samples were irradiated for between 20 min and 11 h before measurement using SAXS and *in situ* pseudo-DSC while heating from −20 to 150 °C. Thermotropic LC phases were first investigated for C_8_AzoC_4_E_4_. After UV irradiation, at −18 °C, a smectic LC with a shorter interlamellar spacing becomes the dominant mesophase. This is shown by the increase in intensity of a set of Bragg peaks at higher *Q* values than those in the *E* isomer (Figure 7b, peaks 1^†^ and 2^†^). This mesophase is also present in the *E* isomer, but the relative intensity increases with irradiation, and after 11 h, this is the only structure present. The shorter *d* spacing suggests that the mesophase is composed of the majority *Z* isomers, which have a shorter end-to-end molecular length due to the bent azobenzene tail. We note that a further smectic phase is formed at −20 °C, at intermediate irradiation times (Figure 7b, peaks 1* and 2*), as discussed fully in the Supporting Information (Section 11). The *Z-*rich smectic phase remains present at room temperature (21 °C, Figure 7d); however, after 11 h of irradiation, the AzoPS is mostly in an isotropic phase, with only a small structural peak corresponding to remaining smectic packing. The loss of LC order is also seen in POM, where an isotropic black phase replaces the birefringent pattern in the *E* isomer (Figure 7c). Only ∼50% of the AzoPS was measured to have isomerized after 11 h of irradiation (*t*_2_, Figure 6b), suggesting that a high isomerization degree is not necessary for the *L*_α_–*I*_0_ transition. *Ex situ* DSC thermograms support the assignment of an isotropic morphology in the *Z-*rich state, showing no endothermic, melting peak on heating from −20 to 150 °C (Figure 7a). In comparison, the *L*_α_ *–I*_0_ phase change in the *E* isomer is visible on the second-heating cycle of the thermogram from *Z* C_8_AzoC_4_E_4_, showing that thermally induced reverse isomerization is associated with cyclable enthalpic behavior in these materials.

**Figure 7:**
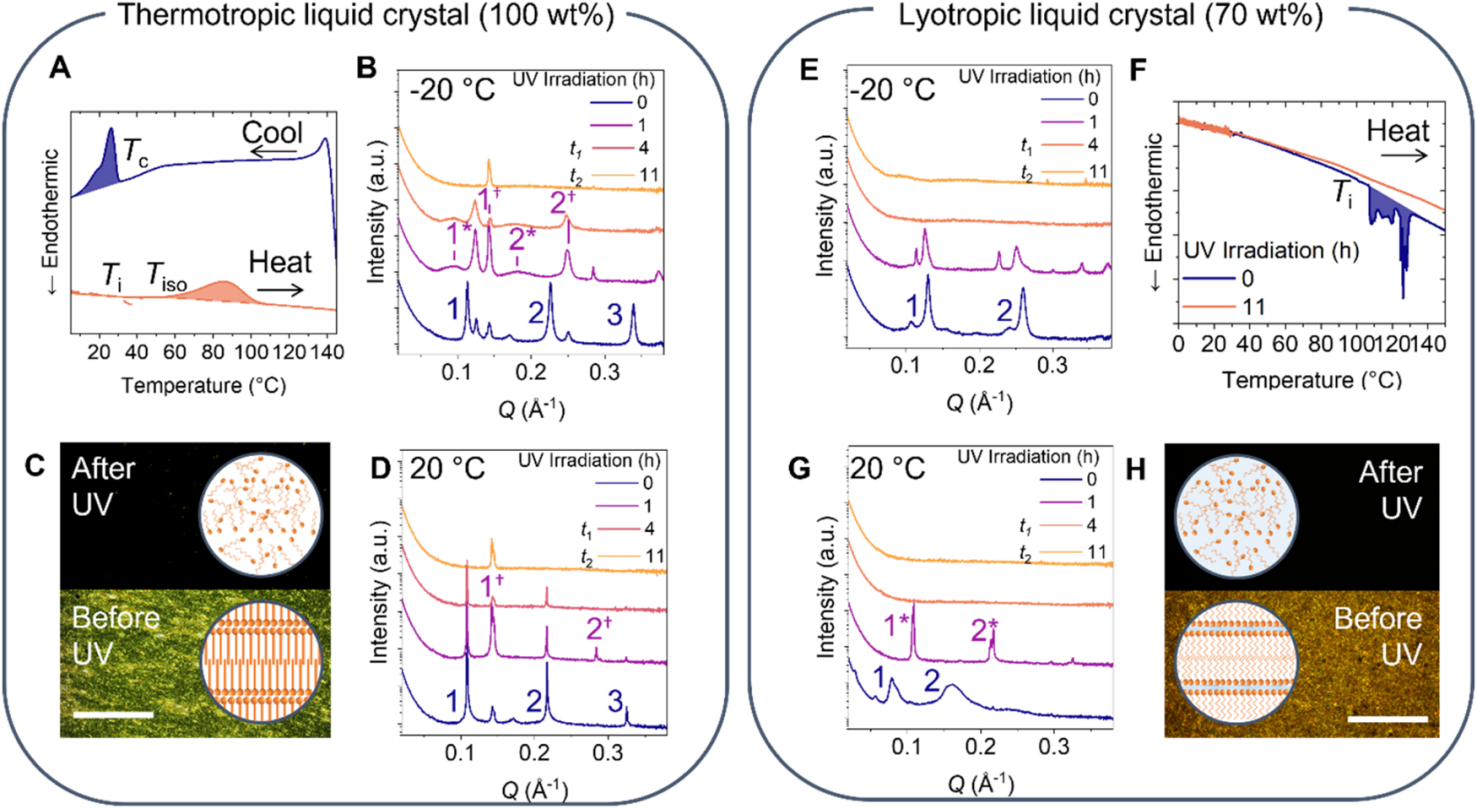
Effect of UV irradiation on thermotropic and lyotropic liquid crystals formed from C_8_AzoC_4_E_4_. In the thermotropic LC phase, (a) DSC thermograms in the *Z*-rich photostationary state show an exothermic, isomerization enthalpy (*T*_iso_) on heating, crystallization enthalpy on cooling (*T*_c_), and clearing enthalpy (*T*_i_) on second heating. SAXS patterns on increasing UV irradiation show progression from a smectic LC to isotropic phases both at (b) −18 and (d) 21 °C, via a series of intermediary smectic arrangements. This is accompanied by (c) a loss of the birefringent pattern under POM. In the lyotropic LC phase, at 70 wt % in water, SAXS patterns show a faster transition from ordered, lamellar to isotropic phases, both at *T*_i_, (e) −18 and (g) 21 °C. This is accompanied by (f) loss of the endothermic, clearing peak *T*_i_ in the *in situ* DSC thermogram and (h) the birefringent POM micrograph, showing a transition to a black, isotropic structure.

We showed earlier that adding solvent to AzoPS can increase the photoisomerization degree, while still facilitating the formation of ordered LLC mesophases. The effect of UV irradiation on the structure of a C_8_AzoC_4_E_4_ LLC (70 wt % in water) was next investigated. From the NMR experiments, we expect isomerization degrees of 2, 12, 40, and 53% for 0, 1, 4 (*t*_1_), and 11 (*t*_2_) hours of UV irradiation. After 1 h, the *L*_α_ peaks in the SAXS data are shifted to higher *Q* values in comparison to the *E* isomer (Figure 7e, peaks 1* and 2*), indicating a shorter interlamellar packing that arises from the formation of the *Z* isomer. Furthermore, irradiation decreases the temperature of the *L*_α_–*I*_0_ transition (from 116 to 60 °C after 1 h), as observed by a loss of the Bragg peaks in the SAXS patterns (see SI, Figure S36), with the sample appearing completely isotropic at all temperatures after 4 h of irradiation (Figure 7). This loss of structural order is also visible using POM (Figure 7h) and through the loss of the endothermic clearing peak in the *in situ* DSC thermogram (Figure 7f, *T*_i_). After the same irradiation time, there was still smectic order in the thermotropic LC, suggesting that the addition of a solvent decreases the irradiation time needed for structural loss. Interestingly, C_8_AzoC_4_E_4_ at concentrations of both 100 and 70 wt % exhibited ∼40% isomerization at this irradiation time (Figure 6b, *t*_1_). The result of irradiation on the more fluid *L*_α_ LLC phase is therefore magnified in comparison to the densely packed, smectic thermotropic LC.

In addition to the phase-change effects, the isomerization enthalpy of AzoPS is important for energy storage. This was determined using *ex situ* DSC measurements and correcting for variation in the isomerization degree using NMR spectroscopy, as described in the Methods Section (Supporting Information). On heating, an exothermic peak is visible for all AzoPS molecules due to *Z–E* isomerization (*T*_iso_, Figures 7a and S43). The maximum theoretical isomerization enthalpy measured varies between 56 and 103 J g^–1^ (33 and 57 kJ mol^–1^) for the different AzoPS molecules (Table 1), showing no trend with the variation in alkyl chain length (Table 1), and are comparable with other azobenzene-based MOST materials.^28^ This was investigated further using density functional theory (DFT) calculations, which predict a decrease in the isomerization enthalpy with an increase in alkyl chain length (from 38.6 to 31.5 kJ mol^–1^ for C_6_AzoC_4_E_4_ to C_10_AzoC_4_E_4_ in aqueous solution, Table 1). This predicted decrease may be due to greater van der Waals’ interactions of the AzoPS on increasing alkyl chain length, which, in turn, reduces the thermodynamic difference between the *E* and *Z* isomers. The discrepancy between the predictions and the experimental measurements suggests that the supramolecular interactions between AzoPS molecules in the solid-state DSC have an essential role in energy storage in these materials.

**Table 1:** Summary of the Isomerization (Δ*H*_iso_), Phase-Change (Δ*H*_phase_), and Total (Δ*H*_tot_) Enthalpy Changes for Thermotropic and Lyotropic LCs of AzoPS at Increasing Alkyl Chain Length (Thermotropic) and Increasing Concentration of C_8_AzoC_4_E_4_ in Water (Lyotropic) at −20 and 20 °C^a^

		Δ*H*_iso_/kJ mol^–1^		Δ*H*_iso_/J g^–1^	Δ*H*_phase_/J g^–1^		Δ*H*_tot_/J g^–1^	
		DSC	DFT		–20 °C	20 °C	–20 °C	20 °C
	AzoPS							
Thermotropic	C_6_AzoC_4_E_4_	46 ± 2	38.6	87 ± 3	17 ± 2	8 ± 1	104 ± 5	95 ± 5
	C_8_AzoC_4_E_4_	57 ± 4	34.4	103 ± 7	35 ± 1	11.2 ± 0.1	138 ± 8	115 ± 7
	C_10_AzoC_4_E_4_	33 ± 4	31.5	56 ± 6	39 ± 2	9 ± 1	95 ± 8	65 ± 7
	Concentration							
Lyotropic	50 wt %			52 ± 7	5 ± 3	1.5 ± 0.0	57 ± 9	50 ± 7
	70 wt %			72 ± 7	3 ± 3	2.6 ± 0.5	76 ± 10	70 ± 7
	90 wt %			93 ± 7	30 ± 5	8.2 ± 1.4	123 ± 12	85 ± 8

a The theoretical isomerization enthalpies given are from DSC thermograms in the solid, *Z*-rich photostationary state and have been corrected for variation in the isomerization change, as measured using NMR spectroscopy. Δ*H*_iso_ from DFT calculations are given in the solution (water) phase. Note that Δ*H*_phase_ at 20 and −20 °C are taken from the first heating cycle of the *E* thermograms. Δ*H*_tot_ at 20 and −20 °C are calculated from the sum of the Δ*H*_iso_ and Δ*H*_phase_ at these temperatures.

For MOST applications, the energy released when reverse isomerization is triggered will be the sum of the isomerization enthalpy and the crystallization enthalpies of the phases formed in the *E* isomer at the operating temperature. On cooling, pure AzoPS exhibits crystallization peaks in the DSC thermograms on reformation of the thermotropic, smectic LC phases present at room temperature (*T*_c_, Figure 7a). It is not possible to measure the crystallization enthalpy of LLC phases using DSC by cooling from the isotropic phase, due to the high temperatures leading to dehydration of the phase, modifying the LLC structure and leading to values that are not representative of the exact concentration that is desired to study. The enthalpy changes for LC formation can therefore be approximated by the phase-change enthalpies on heating. The total enthalpy storage at room temperature (20 °C) and −20 °C can be calculated by summing the isomerization and phase-change enthalpies over the appropriate temperature range (Table 1). For thermotropic LCs, on cooling to 20 °C, there is an additional energy benefit to forming the *L*_α_ phase in all AzoPS. However, the energy storage benefit of the LC phase is greatly enhanced, by up to 30 J g^–1^, on cooling further to −20 °C due to the formation of the crystalline lamellar (*L*_c_) smectic phase, which increases the intermolecular interactions between the AzoPS tail groups. It is worth noting that having additional energy storage benefits on cooling to −20 °C could be beneficial for MOST applications, notably for deicing of motor vehicles,^56^ or thermal regulation in cold climates.^57,58^ The highest stored enthalpy calculated is for C_8_AzoC_4_E_4_, due to its superior isomerization enthalpy, which reached 115 J g^–1^ at room temperature and up to 138 J g^–1^ at −20 °C. Increasing the AzoPS tail length increases the contribution of the phase-change enthalpy to the overall energy storage, with the ratio of phase-change enthalpy-to-isomerization enthalpy at −20 °C increasing from 0.2 to 0.3 to 0.7 for C_6_AzoC_4_E_4_, C_8_AzoC_4_E_4_, and C_10_AzoC_4_E_4_, respectively. Though the isomerization enthalpies used in these calculations are theoretical, the values are competitive with the state-of-the-art, surpassing the highest reported energy-storage density for a liquid crystalline MOST material to date (131 J g^–1^).^32^ However, it falls behind the highest energy densities achieved for phase-change materials (370 J g^–1^) and the MOST target of ∼300 J g^–1^.^21^

For lyotropic LCs, increasing the concentration of AzoPS leads to a greater phase-change enthalpy storage at low temperatures. This is likely due to the formation of more tightly packed phases with greater intermolecular interactions. As in the thermotropic LCs, lowering the temperature to −20 °C similarly increases the energy-storage benefits. This is particularly prevalent at higher AzoPS concentrations, with a total theoretical enthalpy storage of 123 J g^–1^ at −20 °C, or 85 J g^–1^ at 20 °C, associated with the sample at a concentration of 90 wt % (Table 1). LLC systems can therefore achieve competitive energy-storage densities due to the retention of ordered, self-assembled structures on the addition of a solvent. Furthermore, due to the wide range of parameters that can be tuned in these systems, namely, photoswitch, solvent, concentration, and surfactant structure, it is foreseeable that optimization of these systems could boost the energy-storage capacity further.

## Conclusions

In summary, we have demonstrated that AzoPS of three different alkyl chain lengths, C_6_AzoC_4_E_4_, C_8_AzoC_4_E_4_, and C_10_AzoC_4_E_4_, can self-assemble into lyotropic and thermotropic liquid crystal phases with enhanced isomerization and magnified structural control. AzoPS formed smectic, thermotropic LC phases, where the phase transition temperature and enthalpy can be controlled using the alkyl chain length. Upon the addition of water, lamellar (*L*_α_) LLC phases were formed at concentrations of 50 wt % AzoPS or above, which disorder on heating, resulting in an endothermic enthalpy change. When irradiated with UV light, the *E–Z* isomerization degree of the thermotropic LC plateaus at ∼50%; however, this can be increased on addition of water to up to 72% while still retaining an ordered, LLC phase. Not only does the addition of a solvent aid isomerization, but it also amplifies the effects induced at the same isomerization degree. In terms of energy storage, crystallization of the AzoPS into LC phases leads to an exothermic enthalpy change, which would enhance the isomerization enthalpy for MOST systems. Using SAXS with *in situ* DSC for the first time for these systems, we found that, in both thermotropic and lyotropic LCs, while there is an energy-storage benefit to forming the *L*_α_ phase at room temperature, this is greatly enhanced (by up to 30 J g^–1^) on cooling to −20 °C due to the formation of a crystalline lamellar structure with increased intermolecular interactions between the AzoPS tail groups. In the thermotropic system, this could lead to a maximum energy-storage density of 138 J g^–1^. In LLCs, the energy-storage density increases with increasing AzoPS concentration, from 57 to 123 J g^–1^ for concentrations of 50 to 90 wt %. However, this is accompanied by a decrease in the thermal stability of the photoswitch and isomerization degree, meaning that different properties must be balanced for the intended application.

While the introduction of solvent to increase the isomerization degree within azobenzene-based MOST materials has been used frequently in the past, combining this idea with surfactants creates a system where the addition of solvent not only aids isomerization but also drives self-assembly that contributes to the energy storage. In this way, we can achieve systems that are tunable in both structure and isomerization properties by virtue of the solvent. Although the dilution of the AzoPS in LLCs leads to a decrease in the energy density for MOST (achieving up to 123 J g^–1^ cf. 138 J g^–1^ in thermotropic LCs), competitive energy-storage densities are still achieved in both systems. Furthermore, LLCs have the added benefit of faster changes on irradiation, which could lead to a more rapid ‘charging’ time in MOST devices. We have also shown that these LLC phases can be formed in ethylene glycol, which has a lower specific heat capacity and, therefore, is more favorable for MOST applications. While we have used traditional, azobenzene photoswitches in this study, recent work has formed LCs using arylazopyrazole photoswitches, which have greater thermal stability in the *Z* isomer and energy-storage capacities,^47^ and fluoro-functionalized-azobenzene, to achieve visible (rather than UV) light switching, for greater overlap with the solar spectrum.^34^ By combining the new methods used here to unpick structure–isomerization–enthalpy relationships with recent advances in photoswitch optimization, we have created a robust route to liquid crystal optimization for competitive solar energy-storage applications.

## Supplementary Material



## Notes

Materials and experimental methods; synthesis and chemical characterization of AzoPS; calculations for molecular geometry of AzoPS; characterization of AzoPS thermotropic LC mesophases; characterization of AzoPS LLC mesophases; temperature-dependence of AzoPS-water LLCs; characterization of AzoPS-Ethylene glycol LLCs; isomerization studies; characterization of AzoPS-D_2_O LLCs; determination of AzoPS thermal half-life; SAXS with in situ DSC for UV-irradiated AzoPS LCs; DSC thermograms for UV-irradiated AzoPS; and density functional theory calculations (PDF)

## Abbreviations

AzoPS: azobenzene photosurfactant
DMSO: dimethyl sulfoxide
DSC: differential scanning calorimetry
LC: liquid crystal
LLC: lyotropic liquid crystal
MOST: molecular solar thermal energy storage
NMR: nuclear magnetic resonance
POM: polarized optical microscopy
PS: photosurfactant
PSS: photostationary state
SAXS: small-angle X-ray scattering
UV: ultraviolet
