# Supplementary material for: Phase Behavior of Light-Responsive Lyotropic Liquid Crystals for Molecular Solar Thermal Energy Storage
Source: J Am Chem Soc. 2025 Aug 4;147(32):29349–58. doi: 10.1021/jacs.5c09267 (PMC12356579; doi:10.1021/jacs.5c09267)
Supplement: Supplementary file 1 [file ja5c09267_si_001.pdf]

Supporting Information

Phase behavior of light-responsive lyotropic liquid crystals for molecular solar thermal energy storage

*Beatrice E. Jones,[a,b] Zhihang Wang,[a] Martijn A. Zwijnenburg,[c] Charlotte J. C. Edwards-Gayle,[b] Kasper Moth-Poulsen,[d,e,f] Nathan Cowieson[b] and Rachel C. Evans[a]**

[a] Department of Materials Science & Metallurgy, University of Cambridge, 27 Charles Babbage Road, CB3 0FS, United Kingdom

[b] Diamond Light Source, Harwell Science and Innovation Campus, Didcot, Oxfordshire, OX11 0DE, United Kingdom

[c] Department of Chemistry, University College London, 20 Gordon Street, London, WC1H 0AJ, United Kingdom

[d] The Institute of Materials Science of Barcelona, ICMAB-CSIC, Bellaterra, 08193 Barcelona, Spain

[e] Department of Chemical Engineering, Universitat Politècnica de Catalunya, EEBE, Eduard Maristany 10–14, 08019 Barcelona, Spain

[f] Catalan Institution for Research & Advanced Studies, ICREA, Pg. Lluís Companys 23, 08010 Barcelona, Spain

* Corresponding Author: rce26@cam.ac.uk

TABLE OF CONTENTS

1 MATERIALS AND EXPERIMENTAL METHODS 4

1.1 Materials 4

1.2 Sample Preparation 4

1.3 *In-situ* DSC with SAXS 4

1.4 SAXS for LLC characterization 5

1.5 Polarized Optical Microscopy 6

1.6 Differential Scanning Calorimetry 6

1.7 Thermogravimetric Analysis (TGA) 6

1.8 ^{1}H Nuclear Magnetic Resonance Spectroscopy 7

1.9 UV-Vis Absorbance Spectroscopy 7

1.10 Photostability of AzoPS 8

1.11 Density Functional Theory (DFT) calculations 9

2 SYNTHESIS OF AZOPS 9

2.1 Materials 9

2.2 Synthesis of C_mAzoOH (4-alkyl-4'hydroxyl azobenzene, where alkyl = hexyl, octyl or decyl for *m* = 6, 8 and 10 respectively) 10

2.3 Synthesis of bromoprecursor C_mAzoC_4Br (4-alkyl-4'(4-bromo)alkoxy azobenzene, where alkyl = hexyl, octyl or decyl for *m* = 6, 8 and 10 respectively) 10

2.4 Synthesis of C_mAzoC_4E_4 (4-alkyl-4'-(mono-tetraethylene glycol) butoxy azobenzene, replace alkyl with hexyl, octyl or decyl for *m* = 6, 8 and 10 respectively) 10

2.5 Chemical characterization 11

3 ESTIMATING THE MOLECULAR GEOMETRY OF AZOPS 13

4 CHARACTERIZATION OF AZOPS THERMOTROPIC LC MESOPHASES 14

5 CHARACTERIZATION OF AZOPS LLC MESOPHASES 22

6 TEMPERATURE-DEPENDENCE OF AZOPS-WATER LLCS 27

7 CHARACTERIZATION OF AZOPS-ETHYLENE GLYCOL LLCS 32

8 ISOMERIZATION STUDIES 33

9 CHARACTERIZATION OF AZOPS-D_2O LLCS 37

10 DETERMINATION OF AZOPS THERMAL HALF-LIFE 38

11 SAXS WITH *IN-SITU* DSC FOR UV-IRRADIATED AZOPS LCS 41

12 DSC THERMOGRAMS FOR UV-IRRADIATED AZOPS 42

13 DENSITY FUNCTIONAL THEORY 44

14 REFERENCES 47

1 Materials and Experimental Methods

1.1 Materials

Three different types of AzoPS were synthesized ($C_6AzoC_4E_4$, $C_8AzoC_4E_4$, and $C_{10}AzoC_4E_4$) using a method from previous literature,[15] modified for $C_{10}AzoC_4E_4$, as detailed in the Section 2. Products were characterized using ^1H and ^{13}C nuclear magnetic resonance (NMR) spectroscopy and mass spectrometry. Dimethyl sulfoxide-d_6 (DMSO-d_6, 99.5 atom% D), deuterium oxide (D_2O, 99.9 atom% D), and deuterated chloroform ($CDCl_3$, 99.8 atom% D) were purchased from Sigma Aldrich. Ethylene glycol (95%) was purchased from Fisher Scientific. All solvents were analytical grade and were used as received. Water was obtained from a Millipore Simak 2 water purification system.

1.2 Sample Preparation

LLC phases were formed by heating the constituent components ($C_6AzoC_4E_4$, $C_8AzoC_4E_4$ or $C_{10}AzoC_4E_4$ with either water, D_2O or ethylene glycol) to 60°C whilst stirring, until well mixed. Samples were then cooled to room temperature before measurement. The AzoPS concentration was varied with respect to the solvent (10 – 90 wt%). For photoisomerization, samples were irradiated using a custom-built LED light box with UV (365 nm) radiation at an irradiance of 8 W m^{-2} (DSC pan) or 12.4 W m^{-2} (cuvette or NMR tube).

1.3 *In-situ* DSC with SAXS

SAXS heating scans with *in-situ pseudo*-DSC measurements were performed at beamline BM26, at the European Synchrotron Research Facility (Grenoble, France). The X-ray beam energy was 12 keV and the camera length was 3 m. Samples (~5 mg) were loaded into aluminum DSC pans and hermetically sealed before loading into a Linkam DSC600 for measurement. The temperature was controlled using a T96 controller and LINK software, with cooling using liquid nitrogen. For the charging studies, samples were irradiated from the top in the DSC pan for a known time before sealing and measuring. X-ray exposure time was 20 s for every sample, with frames taken every 30 – 60 s during heating cycles. Heating cycles varied slightly depending on the sample, to maximize the beamtime available, whilst still measuring all phase transitions. For 100 wt% samples, cycle 1 was used; for 50 – 90 wt% LLC samples, cycle 2 was used; for UV irradiated samples, cycle 3 was used (Table S1).

Table S1. DSC parameters used during *in-situ* SAXS and DSC studies, where rate is the heating or cooling rate, T_{start} and T_{end} are the start and end temperatures of that ramp and the dwell time is the wait time between reaching the final temperature and ending measurement.

Cycle	Step	Rate / °C min^{-1}	T_{start}/ °C	T_{end}/ °C	Dwell time / min
1	1	5	-20	80	0
	2	2	80	150	0
	3	75	150	25	20
2	1	5	-20	150	0
	2	75	150	25	0
3	1	5	-20	150	0
	2	75	150	25	15

SAXS images were reduced to 1D curves using the processing functionality in DAWN[52] including calibration relative to a silver behenate standard, normalization to measured beam intensity on a beamstop mounted photodiode, definition of Poisson errors and solid angle correction before azimuthal integration. For phase analysis, peak positions were found by subtracting the background and using the Find Peaks tool in Origin(Pro), Version 2021b, OriginLab Corporation. Interlamellar spacings, *d*, were calculated using the position of the primary SAXS peak, Q_0, using the relationship: $d = 2\pi / Q_0$.[2]

1.4 SAXS for LLC characterization

All other SAXS measurements were performed at the high-throughput SAXS beamline B21, Diamond Light Source (Oxfordshire, UK).[4] The X-ray beam energy was 13.1 keV and detector distance set to 3.7 m, giving a *Q* range of 0.0045 – 0.34 Å^{-1}. Samples were loaded into polyimide capillaries (low viscosity) or Kapton tape. Background of water, or ethylene glycol, in polyimide capillary or Kapton tape alone were subtracted using ScÅtter and 20 frames of 1 s exposure time were averaged to give the final data pattern. Unless otherwise stated, samples were measured at 25°C. For temperature ramp samples, the time needed to equilibrate the sample to the given temperature was measured using a thermocouple. The sample was then held at the temperature for this equilibration time before taking a SAXS measurement. Peak fitting and *d* spacing calculations were carried out as above.

1.5 Polarized Optical Microscopy

POM images were taken using a Leica EC3 camera fitted to an Olympus BHM microscope. Samples were placed between 2 glass slides and observed at room temperature under crossed polars. For heating scans, slides were placed on a Linkam PE120 Peltier heat stage controlled by a T96 LinkPad controller and cooled using a water circulation pump.

1.6 Differential Scanning Calorimetry

DSC scans were taken using a Mettler Toledo DSC1 with a liquid nitrogen cooling system and Argon purge gas. Analysis was conducted in the STARe software. Due to the variation in peak positions between samples, peak integration limits were determined by eye and have been included in the results tables as lower and upper limits (°C).

For DSC scans of the *E* isomer in the solid-state, samples (~1 mg) were loaded into aluminum DSC pans and sealed with a pierced lid. Samples were equilibrated at -20°C for 2 minutes, heated from -20 – 150°C at 5 °C min^{-1} and subsequently cooled from 150 – -20°C at 5 °C min^{-1}. One out of three repeats for each chemically distinct sample was heated a second time.

To determine the isomerization enthalpies of the AzoPS, AzoPS were first dissolved in $CDCl_3$ (~10 mg mL^{-1}) and irradiated at 365 nm in a glass vial, whilst stirring, for 2 – 24 h. An NMR scan was taken to confirm isomerization. The sample was then dried using compressed air, whilst still irradiating, for ~4 h. The sample was then transferred to an aluminum DSC pan (~1 mg), with a hole-punched lid for measurement. The remaining dry sample was re-dissolved in $CDCl_3$ and an NMR was taken to calculate the isomerization degree after the drying process. DSC scan consisted of heating from 0 – 150°C at 6 °C min^{-1}, cooling to 0°C at 40 °C min^{-1} and a second heat from 0 – 150°C at 6 °C min^{-1}. After DSC measurement, the pan was broken open and sample dissolved in $CDCl_3$. An NMR spectrum was taken to determine the change in isomerization degree before and after the DSC measurement. To get the theoretical isomerization enthalpy, the measured enthalpy change on isomerization was divided by the percentage change in isomerization degree before and after the DSC scan, to obtain the theoretical enthalpy change on 100% conversion (see Table S13).

1.7 Thermogravimetric Analysis (TGA)

TGA measurements were performed on a TA Instruments Discovery SDT650. $C_8AzoC_4E_4$ at 50 wt% water was measured, with an initial mass of 9.2 mg. The sample was measured from 0 – 700 ºC at a heating rate of 10 ºC min^{-1}, using nitrogen as a purge gas.

1.8 ^{1}H Nuclear Magnetic Resonance Spectroscopy

To calculate the isomerization degree, spectra were taken using a Bruker 400 MHz spectrometer, over 16 scans. Spectra were analyzed using Topspin 4.1.4. For solution studies, AzoPS were dissolved in DMSO-d_6 (10 mM). The sample (1 mL) was loaded into a quartz NMR tube and irradiated with UV (365 nm) light for 60 min, with NMR spectra taken at periodic time stamps (1, 2, 3, 4, 5, 10, 15, 30, 45 and 60 minutes). After 60 minutes, there was negligible further change to the NMR spectra on irradiation.

All spectra were calibrated to DMSO-d_6 (δ = 2.50 ppm). The positions of the azobenzene peaks characteristic to the *E* and *Z* isomers were determined from the 0 and 60 min UV irradiation spectra. For the *E* these are: δ = 7.85 (doublet, 2H), 7.76 (doublet, 2H), 7.38 (doublet, 2H) and 7.11 (doublet, 2H) ppm. For the *Z* these are: δ = 7.14 (doublet, 2H), 6.83 (singlet, 4H), and 6.74 (doublet, 2H) ppm. The peaks were integrated and the doublet at 7.85 ppm assigned an integral of 2. The singlet at 6.83 ppm was divided by 2 and set as 2 separate values for the error calculations. The doublets at 7.11 ppm (*E*) and 7.14 ppm (*Z*) were excluded from analysis as the overlap means they cannot be deconvoluted. The percentage isomerization was calculated by taking a ratio of the average of the *Z* integrals to the sum of the average of *E* and *Z* integrals and multiplying by 100%. Errors were determined using the standard deviations of the integrals of different peaks within the *E* and *Z* assignments.

To determine the percentage isomerization for AzoPS in LCs (50, 70, 90 and 100 wt% in D_2O), samples were loaded into DSC pans (~7 mg) and top-irradiated using UV light for time between 1 – 24 h. After the irradiation period, the pan was transferred to a vial and DMSO-d_6 (500 µL) added. The sample was shaken to dissolve, transferred to an NMR tube and measured immediately. Percentage isomerization was calculated as above.

1.9 UV-Vis Absorbance Spectroscopy

UV-Vis absorbance spectra were measured using a Perkin Elmer Lambda 750 spectrometer with a slit width of 1 nm and a scan speed of 266.75 nm min^{-1}. Measurements were taken at 1 nm intervals from 700 – 200 nm, using quartz cuvettes with a 10 mm path length. Temperature control was achieved using Perkin Elmer Peltier temperature controller 201.

For cycling experiments, a sample of $C_8AzoC_4E_4$ (2 mL, 50 µM in water) was irradiated for 20 minutes with either UV (365 nm) or blue (455 nm) before measurement. This was repeated for 19 complete UV-blue cycles.

To determine the thermal half-life of the *Z* isomer, solutions of AzoPS in water ($C_8AzoC_4E_4$: 100 µM; $C_6AzoC_4E_4$ and $C_{10}AzoC_4E_4$: 50 µM) were made up and loaded (2 mL) into cuvettes.

A reference sample of water was used for all spectra. The UV-Vis absorbance spectrum in the native, *E* state was first taken at 25°C. The sample was irradiated for 20 minutes under UV light, and the formation of the *Z* PSS was confirmed by taking another spectrum at 25°C. The sample was irradiated for a further 10 minutes whilst the sample cell was heated to the measurement temperature (45 – 65°C). Spectra were then taken every 3 minutes for 80 measurements; except for $C_8AzoC_4E_4$ at 45°C, where spectra were taken every 5 minutes for 72 measurements.

Subtracted data were plotted and the position of the absorption maximum for the *E* π-π* transition (λ_{max}) was found using the using the Find Peaks tool in Origin(Pro), Version 2021b, OriginLab Corporation. The percentage isomerized (%*Z*) was calculated using:

$$\%Z = \frac{A_t - A_0}{A_{PSS} - A_0} \quad (1)$$

where A_0 = absorbance at λ_{max} before UV irradiation, A_{PSS} = absorbance at λ_{max} in the photostationary state and A_t = absorbance at λ_{max} at time *t.* Plots of ln(%Z) against time were then fitted to a straight-line to give the first-order rate parameter (*k*) according to:

$$k\,t = \ln(\%Z) \quad (2)$$

Eyring plots of ln(*k/T*) vs. 1/*T* gave the enthalpy change (Δ*H*) and entropy change (Δ*S*) for isomerization according to:

$$k = \frac{k_B T}{h} \exp\left(\frac{-\Delta H}{RT}\right) \exp\left(\frac{\Delta S}{R}\right) \quad (3)$$

where k_B = Boltzmann's constant, *T* = absolute temperature in Kelvin, *h* = Planck's constant, and *R* = molar gas constant. The thermal half-life at 20°C was determined by extrapolating back from the straight-line plot.

1.10 Photostability of AzoPS

For irradiation stability measurements, $C_8AzoC_4E_4$ (2 mL, 50 μM in water) in water was placed in a quartz cuvette with 10 mm path length. The sample was irradiated with UV light (wavelength = 365 nm, irradiance = 6.00 mW cm^{-2}) constantly, whilst taking UV-Vis absorbance spectra every 30 minutes for 24 hours using an Ocean Optics FX Miniature spectrometer with an integration time of 5 ms. The spectra were plotted using Origin(Pro) (\Version 2021b, OriginLab Corporation) and absorbance maxima were found using the Find Peaks tool. The absorbance maximum for the *Z*-rich PSS (315 nm) was plotted against time

to obtain a gauge for damage to the photoswitch on irradiation, which would result in a gradual decrease in this value over time.

1.11 Density Functional Theory (DFT) calculations

A conformer search was performed for both the *E* and *Z* isomers of each molecule using the GFN2-xTB methods by Bannwarth and co-workers[6] and the CREST code.[8] As GFN2-xTB incorrectly predicts the *Z* isomer to be lowest in energy, unconstrained conformer searches for the *E* isomer would yield *Z* isomers instead. Hence, in the case of the conformer search for the *E* isomer, the torsion angle around the -N-N- bond was kept fixed. The lowest conformer for each isomer from the conformer search were subsequently re-optimized in DFT using B97-3c counterpoise corrected composite method by Brandenburg and co-workers,[10] both in vacuum and in water. In the latter case, the solvation in water was described using the COSMO implicit solvation model (ε_r 80). All DFT calculations were performed using the Turbomole code.[12]

2 Synthesis of AzoPS

Three different types of AzoPS were synthesized ($C_6AzoC_4E_4$, $C_8AzoC_4E_4$, and $C_{10}AzoC_4E_4$) using a method from previous literature,[1] modified for $C_{10}AzoC_4E_4$. The method consists of three steps: (1) synthesis of a hydroxyl precursor, 4-alkyl-4'hydroxylazobenzene, from an alkylaniline starting material; (2) S_N2 reaction to form a bromoprecursor, 4-alkyl-4'-(2-bromo)alkyloxy azobenzene; (3) addition of a tetraethylene glycol head group.

For chemical characterization, nuclear magnetic resonance (NMR) spectra were recorded on a Bruker 400 MHz Neo Prodigy spectrometer over 32 scans (^1H spectra) or 512 scans (^{13}C spectra). Samples (ca. 50 mg) were dissolved in $CDCl_3$ (δ = 7.26 ppm, singlet).

2.1 Materials

4-hexylaniline (98%), 4-octylaniline (99%), 4-decylaniline (98%) and anhydrous potassium carbonate (K_2CO_3, 99%) were purchased from Alfa Aesar. Hydrochloric acid (HCl, 37%), phenol (>98%), sodium nitrite (>99%), sodium hydride (NaH, 60% w/w in mineral oil), 1,4-dibromobutane (99%), sodium carbonate (Na_2CO_3, 99.5%), potassium iodide (KI, >99%), tetraethylene glycol (99.5%) and sodium hydroxide (NaOH, >97%) were purchased from Fisher Scientific. All solvents for reactions were analytical grade and were used as received.

2.2 Synthesis of C_mAzoOH (4-alkyl-4'hydroxyl azobenzene, where alkyl = hexyl, octyl or decyl for *m* = 6, 8 and 10 respectively)

4-alkylaniline (10 mmol; 4-hexylaniline: 1.93 mL; 4-octylaniline: 2.28 mL; 4-decylaniline: 2.33 g) was dissolved in acetone (20 mL) and distilled water (20 mL) and HCl (37.5%, 0.024 mol, 2 mL) added. The resulting solution was kept at 0°C, over ice. Sodium nitrite (10 mmol, 0.69 g) was dissolved in distilled water (2 mL), cooled to 1-2°C and added dropwise to the aniline solution. The solution was stirred to form the diazonium salt and placed in the freezer until required. In a separate round-bottom flask, phenol (10 mmol, 0.94 g), Na_2CO_3 (10 mmol, 1.06g) and NaOH (10 mmol, 0.4 g) were dissolved in distilled water (50 mL) and cooled to 1-3°C. The acidic diazonium salt solution was added dropwise, maintaining the temperature below 8°C. Aqueous NaOH solution (10 mL, 25 mmol L^{-1}) was then added dropwise to maintain a pH of 10. The orange precipitate was filtered and washed with distilled water.

2.3 Synthesis of bromoprecursor C_mAzoC_4Br (4-alkyl-4'(4-bromo)alkoxy azobenzene, where alkyl = hexyl, octyl or decyl for *m* = 6, 8 and 10 respectively)

C_mAzoOH (1 eq., 10 mmol: C_6AzoOH: 2.82 g; C_8AzoOH: 3.1 g; C_{10}AzoOH: 3.38 g) was dissolved in acetone (60 mL). 1,4-dibromobutane (2 eq., 20 mmol, 2.39 mL), K_2CO_3 (2 eq., 20 mmol, 2.76 g) and KI (0.1 eq., 1 mmol, 0.17 g) were added and the resulting solution stirred under reflux at 56°C for 48 hours. Acetone was removed *via* rotary evaporation and the product dissolved in dichloromethane (DCM, 20 mL) and washed with water to remove the co-salt. DCM was removed *via* rotary evaporation and C_mAzoC_4Br recovered as an orange solid on recrystallisation with hot ethanol.

2.4 Synthesis of C_mAzoC_4E_4 (4-alkyl-4'-(mono-tetraethylene glycol) butoxy azobenzene, replace alkyl with hexyl, octyl or decyl for *m* = 6, 8 and 10 respectively)

Tetraethylene glycol (5 eq., 25 mmol, 4.81 mL) was dissolved in THF (50 mL) and dried under 3 Å molecular sieves twice. The solution was transferred to a 100 mL round-bottom flask under an inert N_2 atmosphere using a Schlenk line. NaH (60% in oil (w/w), 1.5 eq., 7.5 mmol, 0.3 g) was added and left to stir for 20 minutes. Bromoprecursor (1 eq., 5 mmol, C_6AzoC_4Br: 2.09 g; C_8AzoC_4Br: 2.22 g; C_{10}AzoC_4Br: 2.37 g) was dissolved in dry THF (10 mL) and added to the flask. The solution was stirred under reflux at 65°C for 24 h. Acetone was removed *via* rotary evaporation and the product dissolved in DCM (20 mL) and washed with distilled water. DCM

was removed *via* rotary evaporation and excess bromoprecursor removed using a silica plug with DCM. The product was recovered with acetone and solvent removed to yield C_mAzoC_4E_4 as a red oil.

2.5 Chemical characterization

2.5.1 C_6AzoC_4E_4

^{1}H NMR ($CDCl_3$, 400 MHz, 25°C): *δ* = 7.88 (d, J = 8.9 Hz, 2H), 7.79 (d, J = 8.1 Hz, 2H), 7.29 (d, J = 8.2 Hz, 2H), 6.99 (d, J = 8.9 Hz, 2H), 4.07 (q, J = 6.0 Hz, 2H), 3.71 (t, J = 5.2 Hz, 2H), 3.68 – 3.52 (m, 10H), 2.66 (t, J = 7.3 Hz, 2H), 1.95 – 1.84 (m, 2H), 1.78 (m, J = 14.2, 6.5 Hz, 2H), 1.64 (m, J = 8.1, 7.6 Hz, 2H), 1.40 – 1.22 (m, 6H), 0.89 (t, J = 6.9 Hz, 3H) ppm.

^{13}C NMR ($CDCl_3$, 101 MHz, 25°C): *δ* = 161.4, 151.0, 146.9, 145.8, 129.0, 124.6, 122.5, 114.7, 72.8, 71-70 (7 peaks), 68.0, 62.5, 61.7, 35.9, 31.7, 31.3, 31.0, 29.3, 29.0, 26.1, 26.0, 25.7, 22.6, 14.1 ppm.

Mass Spectrometry (CH_3CN, *m/z* APCI$^+$): For $[M+H]^+$ exact mass calculated: 530.3356; found: 530.3356.

Yield: 65%

2.5.2 C_8AzoC_4E_4

^{1}H NMR ($CDCl_3$, 400 MHz, 25°C): *δ* = 7.88 (d, J = 8.9 Hz, 2H), 7.79 (d, J = 8.3 Hz, 2H), 7.29 (d, J = 8.2 Hz, 2H), 6.98 (d, J = 8.9 Hz, 2H), 4.06 (t, J = 6.3 Hz, 2H), 3.75 – 3.45 (m, 14H), 2.66 (t, J = 7.7 Hz, 3H), 1.95 – 1.84 (m, 2H), 1.84 – 1.73 (m, 2H), 1.70 – 1.57 (m, 2H), 1.41 – 1.15 (m, 12H), 0.87 (t, J = 6.8 Hz, 3H) ppm.

^{13}C NMR ($CDCl_3$, 101 MHz, 25°C): *δ* =161.46, 151.11, 147.03, 145.91, 129.14, 124.65, 122.79, 114.78, 72.88, 71-70 (5 peaks), 68.08, 61.76, 35.96, 31.98, 31.42, 31.04, 29.57, 29.36, 26.09, 22.77, 14.22 ppm.

Mass Spectrometry (CH_3CN, *m/z* APCI$^+$): For $[M+H]^+$ exact mass calculated: 558.3669; found: 558.3681.

Yield: 42%

2.5.3 C_{10}AzoC_4E_4

^{1}H NMR ($CDCl_3$, 400 MHz, 25°C): *δ* = 7.88 (d, J = 8.9 Hz, 2H), 7.79 (d, J = 8.3 Hz, 2H), 7.29 (d, J = 8.3 Hz, 2H), 6.99 (d, J = 8.9 Hz, 2H), 4.06 (t, J = 6.3 Hz, 2H), 3.71 (t, J = 4.2 Hz, 2H), 3.67 – 3.52 (m, 14H), 2.66 (t, J = 8.7 Hz, 2H), 1.94 – 1.84 (m, 2H), 1.84 – 1.73 (m, 2H), 1.69 – 1.58 (m, 2H), 1.38 – 1.17 (m, 16H), 0.87 (t, J = 6.7 Hz, 3H) ppm.

^{13}C NMR ($CDCl_3$, 101 MHz, 25°C): δ = 161.47, 151.12, 147.05, 145.92, 129.15, 124.68, 122.61, 115.53, 72.91, 71-70 (6 peaks), 68.09, 61.77, 35.97, 32.01, 31.46, 31.06, 29.73, 29.70, 29.61, 26.10, 22.80, 14.24 ppm.

Mass Spectrometry (CH_3CN, m/z: $APCI^+$): Exact mass calculated: 586.3982. Exact mass obtained: 586.3998.

Yield: 51%

3 Estimating the molecular geometry of AzoPS

The critical packing parameter (*CPP*) for an amphiphile can be used to predict the lyotropic liquid crystal phase it is likely to form due to the spontaneous curvature favored by the self-assembled amphiphiles. The *CPP* is defined as: $CPP = v/a_0l_c$, where v is the volume of the hydrophobic tail, a_0 is the hydrophilic head group area and l_c is the length of the hydrophobic chain.[3] To calculate the packing parameter for AzoPS, v and l_c were determined using the empirical volume additivity rule of Traube:[5]

$$v = \sum v_i \tag{4}$$

$$l_c = \sum l_{ci} \tag{5}$$

where v_i and l_{ci} are the contributions of the ith components for the tail volume and length, respectively.

The Tanford equations are commonly used to calculate the lengths and volumes of alkyl chain of surfactants[7] and were used to obtain the contributions of the alkyl chain of AzoPS.

$$v = 26.9 \times m + 27.4 \tag{6}$$

$$l_c = 1.265 \times m + 1.5 \tag{7}$$

where m is the number of $-CH_2-$ carbons in the carbon chain. Further contributions to v and l_c were found using literature values from various methods as summarized in Table S2. The contributions were added to give estimates for v, a_0 and l_c for the three different AzoPS structures in the *E* isomeric state (Table S3). These were used to calculate the *CPP*.

Table S2. Contribution of volumes, lengths and head-group areas to calculate the critical packing parameter, *CPP*, for AzoPS in the *E* isomeric state using the additivity rule.

Component	Value	Method
Alkyl chain volume (nm^3)	0.0269m + 0.0274	Tanford equations[7]
E azobenzene volume (nm^3)	0.176	MOPAC calculations, van der Waals volume [9]
Oxy-group volume (nm^3)	9.1×10^{-3}	*Ab initio* calculations, van der Waals volume[5]
Alkyl chain length (nm)	0.1265m +0.15	Tanford equations[7]
E azobenzene length (nm)	0.9	X-ray analysis[11]
Oxy-group length (nm)	0.28	DFT calculations[13]
E_4 head-group area (nm^2)	0.46	Surface tension study of $C_{12}E_4$ at 25°C[14]

Table S3. Amphiphile geometrical parameters as calculated for AzoPS in the *E* state. The volume of the hydrophobic tail, *v*; hydrophilic head group area, a_0; and length of the hydrophobic chain, l_c, contribute to the critical packing parameter, *CPP*.

AzoPS	*v* (nm^3)	a_0 (nm^2)	l_c (nm)	*CPP*
$C_6AzoC_4E_4$	0.48	0.46	2.62	0.40
$C_8AzoC_4E_4$	0.54	0.46	2.87	0.41
$C_{10}AzoC_4E_4$	0.60	0.46	3.12	0.42

4 Characterization of AzoPS thermotropic LC mesophases

For all AzoPS structures, the thermotropic LC phase formation and temperature-dependence was determined using SAXS with *in-situ* DSC and POM. $C_6AzoC_4E_4$ forms smectic LC phases from -18 – 100°C, as shown by the formation of Bragg peaks in the SAXS with *Q* positions in a ratio of 1:2:3 (Figure S1) and a birefringent, striped pattern using POM (Figure S2). An increase in the interlamellar (*d*) spacing at -3°C indicates a crystalline (L_c) to fluid (L_α) lamellar rearrangement at this temperature (at T_m, Figure S1, and Table S4). Above 100°C (the clearing temperature, T_i), the Bragg peaks in the SAXS are lost (Figure S1), indicating a L_α-isotropic (I_0) transition, which is accompanied by an endothermic enthalpy change in the *in-situ* DSC (Figure S3). The L_α-I_0 transition is also visible as a loss of the birefringent pattern in the POM (Figure S2), but this occurs at a lower temperature of 50°C.

The thermotropic LC phase formation for $C_8AzoC_4E_4$ is discussed in the main text. We note that for this structure, the SAXS shows an additional phase transition at -18°C, shown by

a shift in the d spacing (Table S5). This could be due to relaxation of the AzoPS to their equilibrium structure on cooling to the start temperature and is not associated with any enthalpy changes in either *in-* or *ex-situ* DSC. We also note that, whilst POM shows loss of the striped texture from 60°C (in agreement with the phase transition in SAXS, Figure S2), some bright, crystalline textures remain in the sample until 110°C, which indicate the presence of heterogenous crystallites.

$C_{10}AzoC_4E_4$ also forms smectic LC mesophases from -18 – 86°C, as seen using SAXS and POM (Figures S1 and S2). A L_c-L_α phase transition occurs at 16°C, as seen by an increase in d spacing at this temperature (Table S6), accompanied by an endothermic peak in the *in-situ* DSC (Figure S3). The L_α-I_0 transition was observed at 86°C using SAXS, accompanied by an endothermic peak in the DSC (Figures S1 and S3). Using POM, the L_α-I_0 transition was observed at a slightly lower temperature of ~70°C (Figure S2).

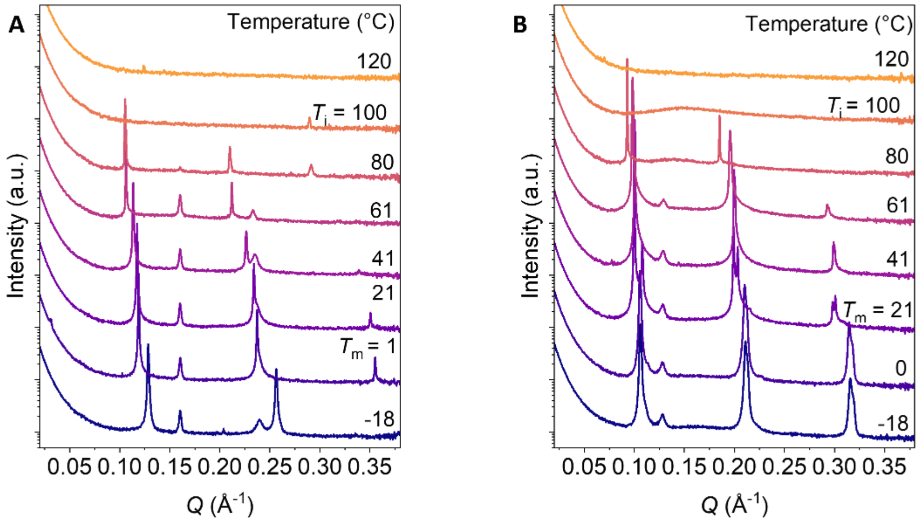

Figure S1. SAXS patterns for (a) $C_6AzoC_4E_4$ and (b) $C_{10}AzoC_4E_4$ on heating from -18 to 120°C showing the formation of ordered, smectic phases, evidenced by the formation of Bragg peaks. A lamellar crystalline (L_c)-to-fluid (L_α) transition occurs at the melting temperature (T_m), resulting in a shift of the Bragg peaks to lower Q values. A smectic-to-isotropic (I_0) transition occurs at the clearing temperature (T_i), resulting in a loss of the peaks.

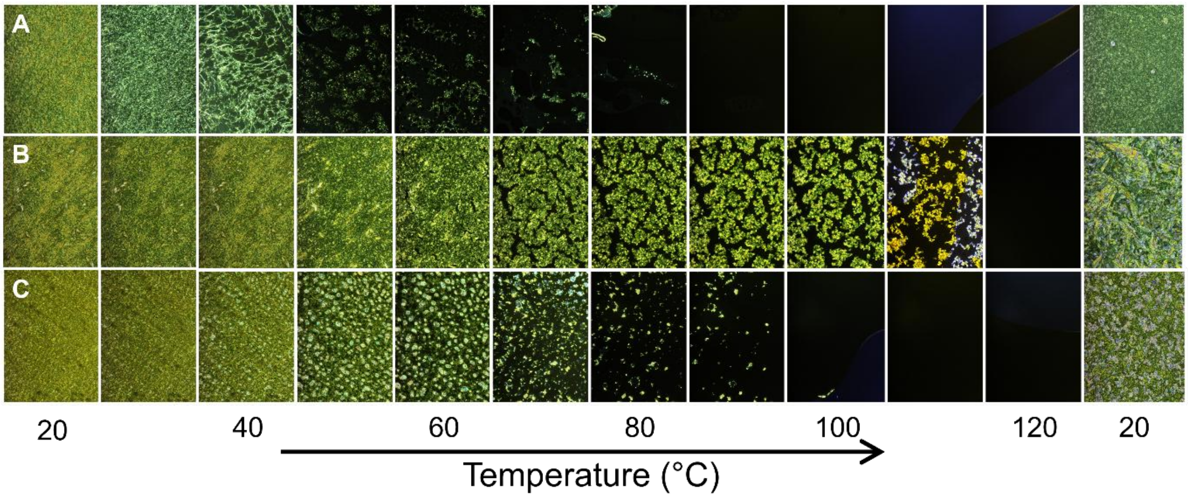

Figure S2. Polarized optical micrographs from a heat scan for AzoPS (100 wt%) of increasing alkyl chain length, (a) $C_6AzoC_4E_4$ (b) $C_8AzoC_4E_4$, (c) $C_{10}AzoC_4E_4$, on heating between 20 – 120°C and on cooling back to 20°C. Bright, striped patterns at lower temperatures indicate the presence of a smectic LC phases, where black micrographs indicate an isotropic phase.

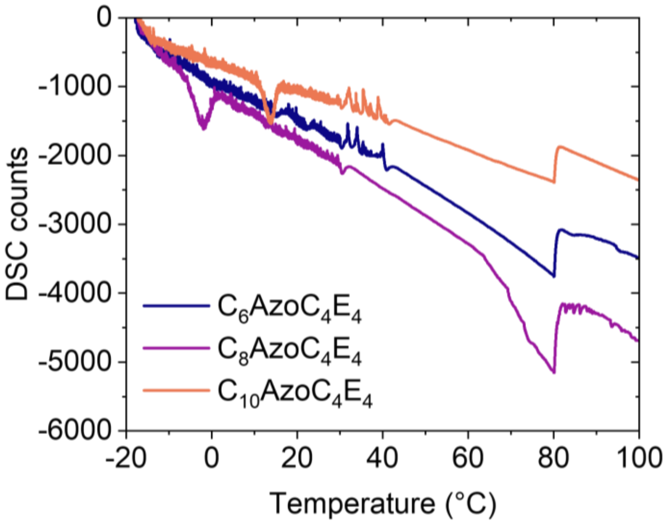

Figure S3. *In-situ pseudo*-DSC thermograms during SAXS for AzoPS of increasing alkyl chain length ($C_6AzoC_4E_4$, $C_8AzoC_4E_4$, and $C_{10}AzoC_4E_4$) on heating from -20 to 100°C.

Table S4. Positions of the Bragg peaks in the SAXS curves for $C_6AzoC_4E_4$ at increasing temperature. The peaks have been sorted into those assigned to a primary and secondary lamellar phase, with the lamellar spacing (d_1 and d_2) calculated using the position of the primary peak for each phase. Above 100°C the scattering curve contains no Bragg peaks, indicating an isotropic phase.

Temperature	Peak positions - primary (Å^{-1})			Peak positions - secondary (Å^{-1})			*d* spacing (nm)	
(°C)	Q_0	Q_1	Q_2	Q_0	Q_1	Q_2	d_1	d_2
-18	0.1286	0.2562	0.3842				4.89	
-13	0.1279	0.2559	0.3829				4.91	
-8	0.1276	0.2550	0.3814				4.92	
-3	0.1189	0.2371	0.3560	0.1270	0.2537	0.3804	5.29	4.95
1	0.1189	0.2371	0.3553				5.29	
6	0.1185	0.2368	0.3544				5.30	
11	0.1182	0.2362	0.3535				5.31	
16	0.1179	0.2352	0.3522				5.33	
21	0.1173	0.2340	0.3506				5.36	
26	0.1167	0.2327	0.3484				5.39	
31	0.1157	0.2311	0.3459				5.43	
36	0.1148	0.2289	0.3428				5.47	
41	0.1135	0.2264	0.3390				5.54	
46	0.1116	0.2233					5.63	
51	0.1060	0.2114		0.1091	0.2189		5.93	5.76
56	0.1060	0.2117					5.93	
61	0.1063	0.2123					5.91	
66	0.1063	0.2123					5.91	
71	0.1060	0.2123					5.91	
76	0.1060	0.2117					5.93	
80	0.1054	0.2101					5.96	
86	0.1029	0.2054					6.11	
90	0.2904						2.16	
96	0.2901						2.17	
100	0.2895						2.17	
106 - 149				Isotropic				

Table S5. Positions of the Bragg peaks in the SAXS curves for $C_8AzoC_4E_4$ at increasing temperature. The peaks have been sorted into those assigned to a primary and secondary lamellar phase, with the lamellar spacing (d_1 and d_2) calculated using the position of the primary peak for each phase. Above 80°C the scattering curve contains no Bragg peaks, indicating an isotropic phase.

Temperature	Peak positions - primary (Å^{-1})			Peak positions - secondary (Å^{-1})			d spacing (nm)	
(°C)	Q_0	Q_1	Q_2	Q_0	Q_1	Q_2	d_1	d_2
-18	0.1135	0.2264	0.3390	0.1254	0.2500		5.54	5.01
-13	0.1135	0.2261	0.3384				5.54	
-8	0.1132	0.2258	0.3384				5.55	
-3	0.1132	0.2258	0.3381				5.55	
1	0.1098	0.2189	0.3277				5.72	
6	0.1094	0.2186	0.3274				5.74	
11	0.1094	0.2183	0.3268				5.74	
16	0.1094	0.2176	0.3259				5.76	
21	0.1088	0.2170	0.3249				5.77	
26	0.1085	0.2164	0.3240				5.79	
31	0.1082	0.2158	0.3230				5.81	
36	0.1076	0.2148	0.3218				5.84	
41	0.1072	0.2139	0.3199				5.86	
46	0.1063	0.2120	0.3174				5.91	
51	0.1047	0.2095					6.00	
56	0.0966						6.51	
61	0.0960						6.55	
66	0.1452						4.33	
71	0.1452						4.33	
76	0.1449						4.34	
80 - 149				Isotropic				

Table S6. Positions of the Bragg peaks in the SAXS curves for $C_{10}AzoC_4E_4$ at increasing temperature. The peaks have been sorted into those assigned to a primary and secondary lamellar phase, with the lamellar spacing (d_1 and d_2) calculated using the position of the primary peak for each phase. Above 86°C the scattering curve contains no Bragg peaks, indicating an isotropic phase.

Temperature	Peak positions - primary (Å^{-1})			Peak positions - secondary (Å^{-1})			d spacing (nm)	
(°C)	Q_0	Q_1	Q_2	Q_0	Q_1	Q_2	d_1	d_2
-18	0.1060	0.2111	0.3158				5.93	
-15	0.1060	0.2111	0.3158				5.93	
-10	0.1057	0.2107	0.3155				5.95	
-5	0.1057	0.2104	0.3152				5.95	
0	0.1054	0.2101	0.3146				5.96	
6	0.1054	0.2098	0.3142				5.96	
11	0.1051	0.2098	0.3139				5.98	
16	0.0994	0.2029	0.2998	0.1088	0.2167		6.32	5.77
21	0.0997	0.1991	0.2979				6.30	
26	0.1003	0.2001	0.2995				6.26	
31	0.1003	0.2004	0.2998				6.26	
36	0.1003	0.2001	0.2995				6.26	
41	0.1003	0.1998	0.2992				6.26	
46	0.1000	0.1991	0.2983				6.28	
51	0.0994	0.1985	0.2970				6.32	
56	0.0991	0.1973	0.2954				6.34	
61	0.0982	0.1957	0.2929				6.40	
66	0.0969	0.1929	0.2885				6.48	
70	0.0919	0.1831					6.84	
75	0.0922	0.1841					6.82	
80	0.0928	0.1850					6.77	
86	0.0931	0.1857					6.75	
86 - 149				Isotropic				

The quantitative values for the enthalpy changes associated with phase transitions were determined using *ex-situ* DSC measurements. Three separate scans were taken on identical samples to test reproducibility (Figure S4), and results for peak integrations were averaged with errors given by standard deviation (Table S7). It should be noted that T_m and T_i vary depending on the measurement technique, with higher temperatures required for structure loss in SAXS than measured for endothermic peaks in DSC and POM (~50°C in DSC *cf.* 80°C in SAXS). This could be due to different loading requirements for each technique (e.g. a higher mass is needed to acquire enough scattering contrast for SAXS, and thinner samples are pinned by the glass slides for POM), which may have a large effect on these materials, due to their interface-specific nature as surfactants. It should also be noted that for $C_8AzoC_4E_4$, in the DSC scan, there is a small exothermic peak before the endothermic peak associated with the L_c-L_α phase transition (Figure S4b), which could be due to chain rearrangement before fluidization. To determine that the DSC scans occur below the AzoPS degradation temperature, TGA analysis was also undertaken. $C_8AzoC_4E_4$ was measured at 50 wt% in water, showing initial mass loss to 58 wt% at 100 ºC (Figure S5). We note that the mass does not reach 50 wt% on dehydration, which indicates some water is still tightly-bound to the AzoPS above 100 ºC. AzoPS degradation occurs above 250 ºC, meaning the DSC scans shown here all occur below the degradation temperature.

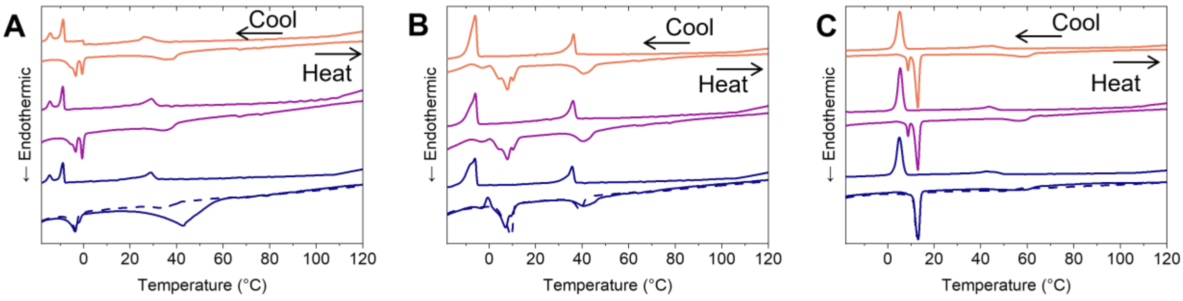

Figure S4. DSC thermograms for three separate samples of AzoPS (100 wt%) on heating, cooling and second heat (dashed line, only measured for one sample) for AzoPS of increasing alkyl chain length (a) $C_6AzoC_4E_4$, (b) $C_8AzoC_4E_4$ and (c) $C_{10}AzoC_4E_4$.

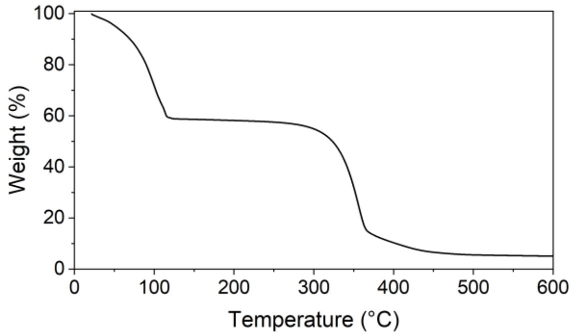

Figure S5. TGA thermogram for $C_8AzoC_4E_4$ (50 wt% in water) on heating. Initial sample dehydration occurs at 100 ºC, with sample degradation onset above 250 ºC.

Table S7. *Ex-situ* DSC data for AzoPS ($C_6AzoC_4E_4$, $C_8AzoC_4E_4$ and $C_{10}AzoC_4E_4$) in the solid-state. The upper and lower temperature limits of integration (°C) and enthalpy changes (ΔH) are given for heating, cooling, and second heating cycles, as an average over three samples, except for the values indicated. Errors are calculated from the standard deviation of these results. The phase changes, as assigned using SAXS have also been given, where L_c = lamellar crystalline smectic, L_α = fluid lamellar smectic, and I_0 = isotropic melt.

AzoPS	Cycle	Lower limit (°C)	Upper limit (°C)	ΔH (J g^{-1})	ΔH (kJ mol^{-1})	Phase change
$C_6AzoC_4E_4$	Heat 1	-12	1	9.0 ± 0.6	4.8 ± 0.3	$L_c \rightarrow L_\alpha$
		23*	44*	8.3 ± 1.4*	4.4 ± 0.7	$L_\alpha \rightarrow I_0$
	Cool	-18	-7	-6.3 ± 0.4	-3.3 ± 0.2	$L_\alpha \rightarrow L_c$
		20	35	-4.6 ± 1.4	-2.4 ± 0.7	$I_0 \rightarrow L_\alpha$
	Heat 2	-10	2	8.6	4.6	$L_c \rightarrow L_\alpha$
		24	43	5.4	2.9	$L_\alpha \rightarrow I_0$
$C_8AzoC_4E_4$	Heat 1	-2†	1†	-2.3†	-1.3	
		0	14	21.5 ± 1.3	12.0 ± 0.7	$L_c \rightarrow L_\alpha$
		30	49	11.2 ± 0.1	6.3 ± 0.1	$L_\alpha \rightarrow I_0$
	Cool	-15	-3	-20.4 ± 1.8	-11.4 ± 1.0	$L_\alpha \rightarrow L_c$
		26	40	-9.5 ± 0.7	-5.3 ± 0.4	$I_0 \rightarrow L_\alpha$
	Heat 2	1	13	21.6	12	$L_c \rightarrow L_\alpha$
		30	43	8.7	4.8	$L_\alpha \rightarrow I_0$
$C_{10}AzoC_4E_4$	Heat 1	4	17	30.6 ± 1.0	17.9 ± 0.6	$L_c \rightarrow L_\alpha$
		44	67	8.6 ± 0.9	5.1 ± 0.6	$L_\alpha \rightarrow I_0$
	Cool	-3	10	-28.8 ± 0.5	-16.9 ± 0.3	$L_\alpha \rightarrow L_c$
		32	53	-5.2 ± 1.9	-3.0 ± 1.1	$I_0 \rightarrow L_\alpha$
	Heat 2	3	17	29.7	17.4	$L_c \rightarrow L_\alpha$
		38	59	4.9	2.8	$L_\alpha \rightarrow I_0$

* Peak only present in two of three runs.
† Peak only present in one of three runs.

5 Characterization of AzoPS LLC mesophases

AzoPS LLC mesophases were characterized using a combination of SAXS and POM. $C_6AzoC_4E_4$ forms swollen lamellar ($L_{\alpha,s}$) phases at concentrations from 10 - 50 wt% in water, shown by the formation of two peaks in the *Q* ratio of 1:2 in the SAXS, associated with a large *d* spacing of 9.2 nm (Figure S6, Table S8). Above 70 wt%, this transitions to a more densely packed L_α phase, with a decrease in *d* spacing from 9.2 to 5.6 nm, and a denser birefringence pattern under POM (Figures S5 and S6). At 70 wt%, multiple different lamellar packings are present, as can be seen by multiple peaks and shoulders in the SAXS pattern (Figure S7d); however, at 90 wt% only one L_α phase dominates.

Table S8. Summary of the LLC mesophases and interlamellar (*d*) spacings formed for all three AzoPS structures ($C_6AzoC_4E_4$, $C_8AzoC_4E_4$, and $C_{10}AzoC_4E_4$) on increasing concentration (wt%) in water. Swollen lamellar ($L_{\alpha,s}$), lamellar (L_α) and isotropic micellar (I_0) mesophases form. In some cases, multiple lamellar phases are present and the *d* spacings for the primary (1°), secondary (2°) and tertiary (3°) mesophases are given.

**AzoPS**	**AzoPS Concentration (wt%)**	**LLC mesophase**	***d* (nm)**		
			**1°**	**2°**	**3°**
$C_6AzoC_4E_4$	10	$L_{\alpha,s}$	9.2	-	-
	30	$L_{\alpha,s}$	9.5	-	-
	50	$L_{\alpha,s}$	8.9	-	-
	70	L_α	8.4	7.7	-
	90	L_α	5.6	-	-
$C_8AzoC_4E_4$	10	I_0	-	-	-
	30	I_0	-	-	-
	50	$L_{\alpha,s}$	10.3	-	-
	70	L_α	7.8	7.5	6.8
	90	L_α	5.8	-	-
$C_{10}AzoC_4E_4$	10	I_0	-	-	-
	30	I_0	-	-	-
	50	$L_{\alpha,s}$	26.6	-	-
	70	L_α	7.4	7.0	6.8
	90	L_α	6.3	-	-

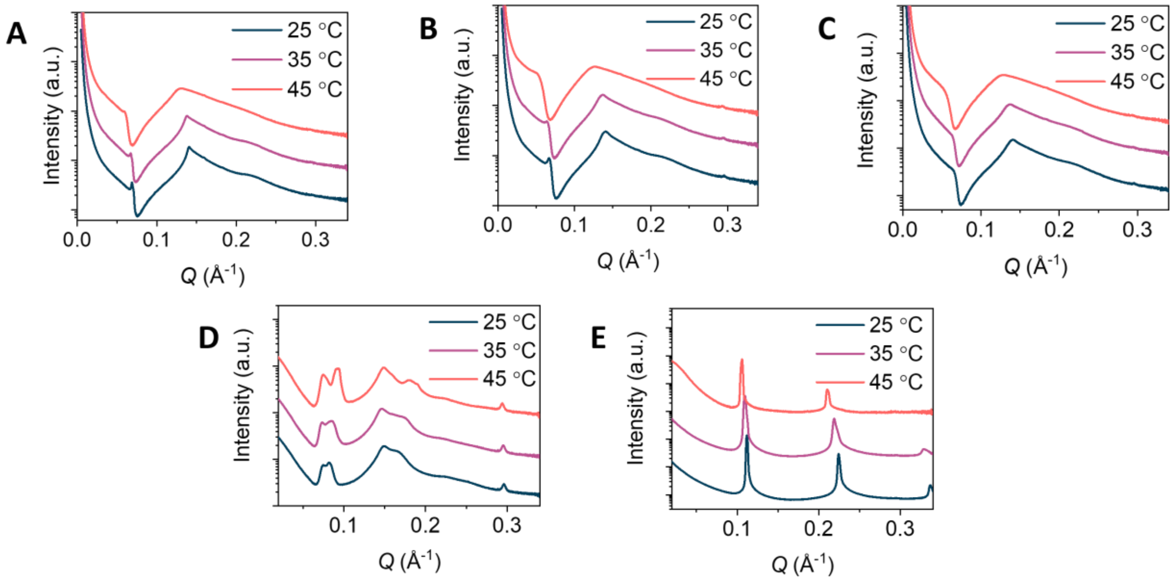

Figure S6. SAXS plots for $C_6AzoC_4E_4$ at increasing temperature in the *E* isomer, for increasing concentrations in water (a) 10 wt%, (b) 30 wt%, (c) 50 wt%, (d) 70 wt% and (e) 90 wt%.

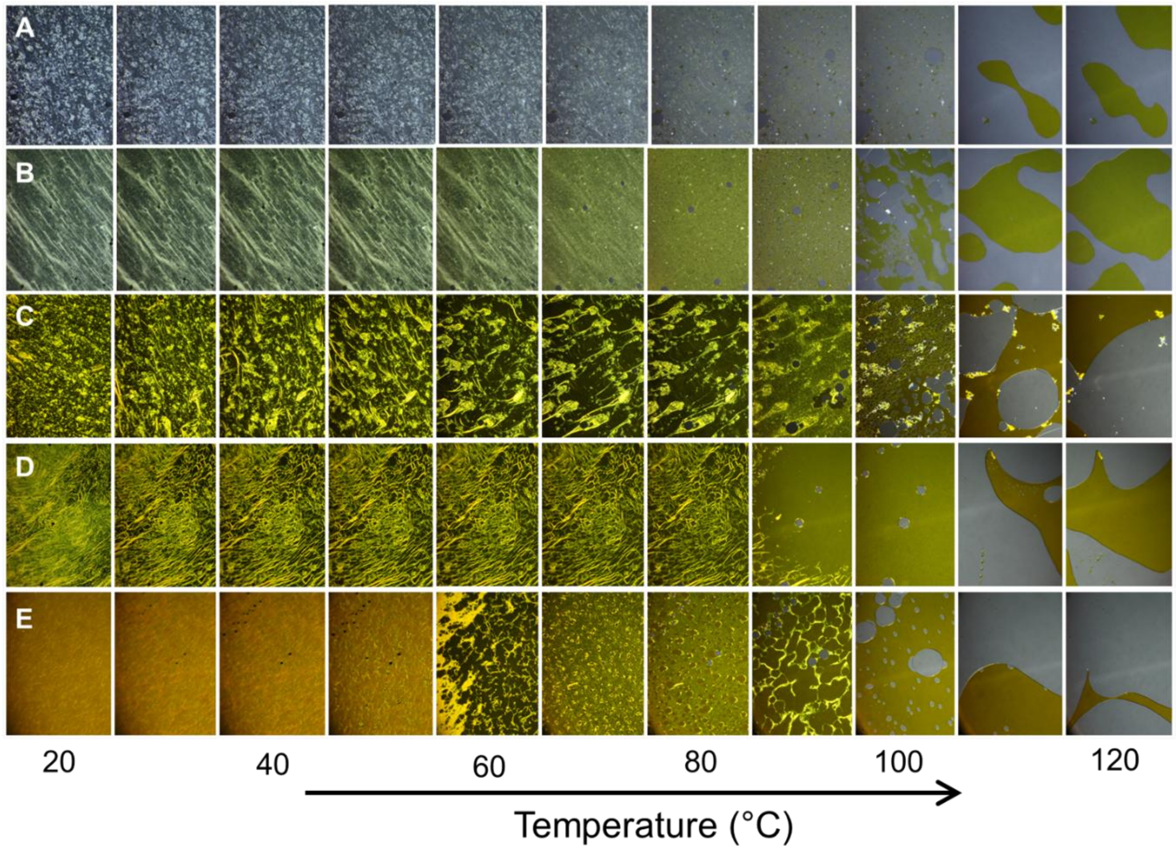

Figure S7. Polarized optical micrographs for $C_6AzoC_4E_4$ in the *E* isomer on heating from 20 - 120°C at increasing concentration (a) 10, (b), 30, (c) 50, (d) 70 and (e) 90 wt% in water.

The characterization of $C_8AzoC_4E_4$-water LLCs is discussed in the main text. We note the formation of a small peak at Q = 0.14 $Å^{-1}$ in the SAXS for 10 – 30 wt% (Figure S8), which can be seen as bright, birefringent crystallites in the POM (Figure S9). At 50 wt%, we have assigned a lamellar mesophase ($L_{α,s}$), with the large d spacing (10.3 nm) suggesting a swollen phase. Previous studies have reported a hexagonal phase for this molecule at 50 wt% in water, with only a small contribution from the $L_α$ phase.[15] This discrepancy could be due to differences in sample loading; previously the sample was auto-loaded into a capillary, whilst here samples were transferred into a cavity in a solid-sample holder, meaning differences in shear at the sample surface may affect the LLC phase. For 70 wt%, the SAXS peaks have shoulder regions, stretching to higher Q values, that can be resolved into contributions from different lamellar phases, of decreasing d spacing, which change in intensity on heating (Figure S8d, Table S8). At 90 wt%, only one $L_α$ is visible in the SAXS.

The longer $C_{10}AzoC_4E_4$ only assembles into LLC phases at 50 wt% and above (Figures S10 and S11), forming I_0 mesophases at 10 and 30 wt%. From 50 – 90 wt% a series of $L_α$ phases form, tending to a more densely-packed morphology with increasing wt% and a decrease in d spacing from 26.6 to 6.3 nm from 50 – 90 wt% (Table S8).

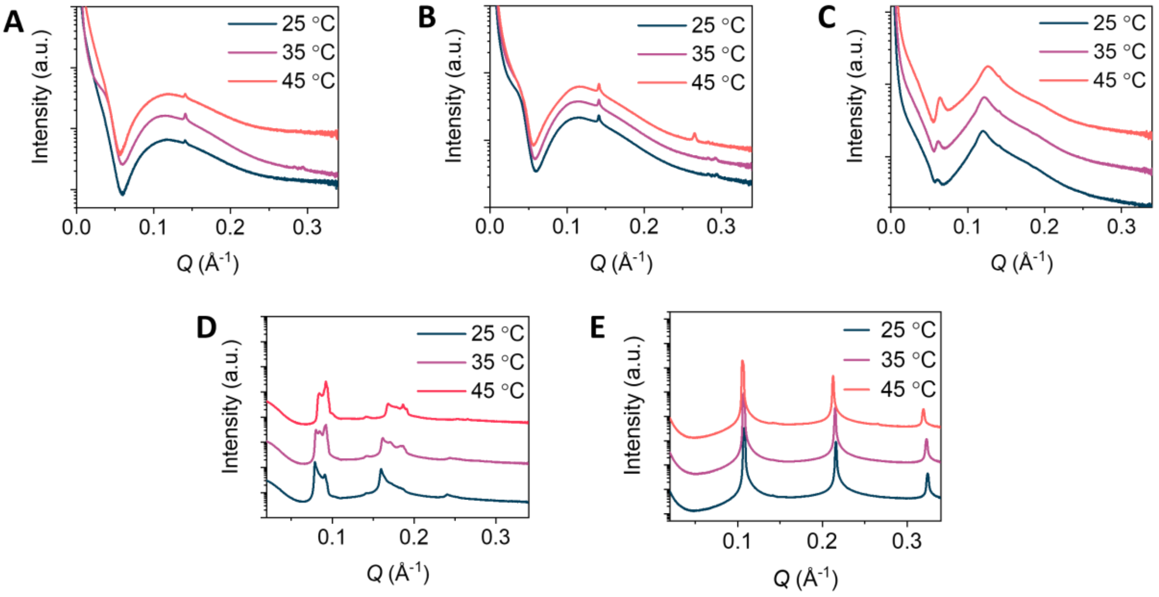

Figure S8. SAXS plots for $C_8AzoC_4E_4$ at increasing temperatures in the *E* isomer, for increasing concentrations in water (a) 10 wt%, (b) 30 wt%, (c) 50 wt%, (d) 70 wt% and (e) 90 wt%.

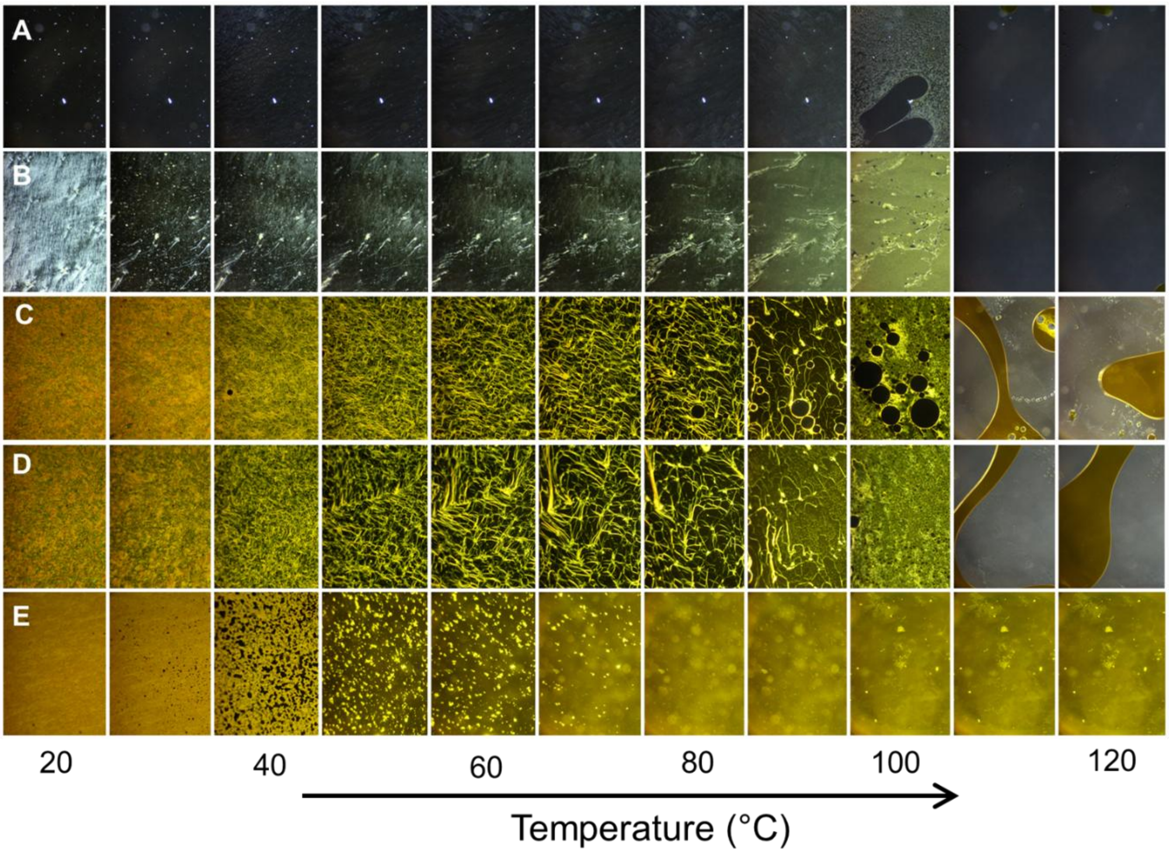

Figure S9. Polarized optical micrographs for $C_8AzoC_4E_4$ at increasing concentrations (wt%) in water, on heating from 20 - 120°C.

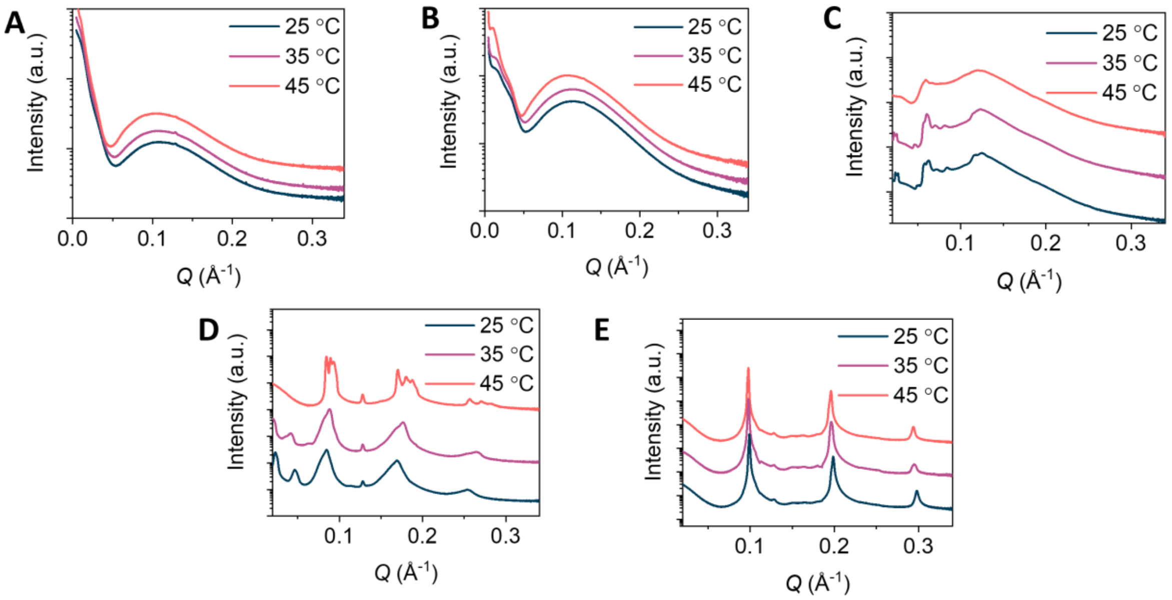

Figure S10. SAXS plots for $C_{10}AzoC_4E_4$ at increasing temperatures in the *E* isomer, for increasing concentrations in water (a) 10 wt%, (b) 30 wt%, (c) 50 wt%, (d) 70 wt% and (e) 90 wt%.

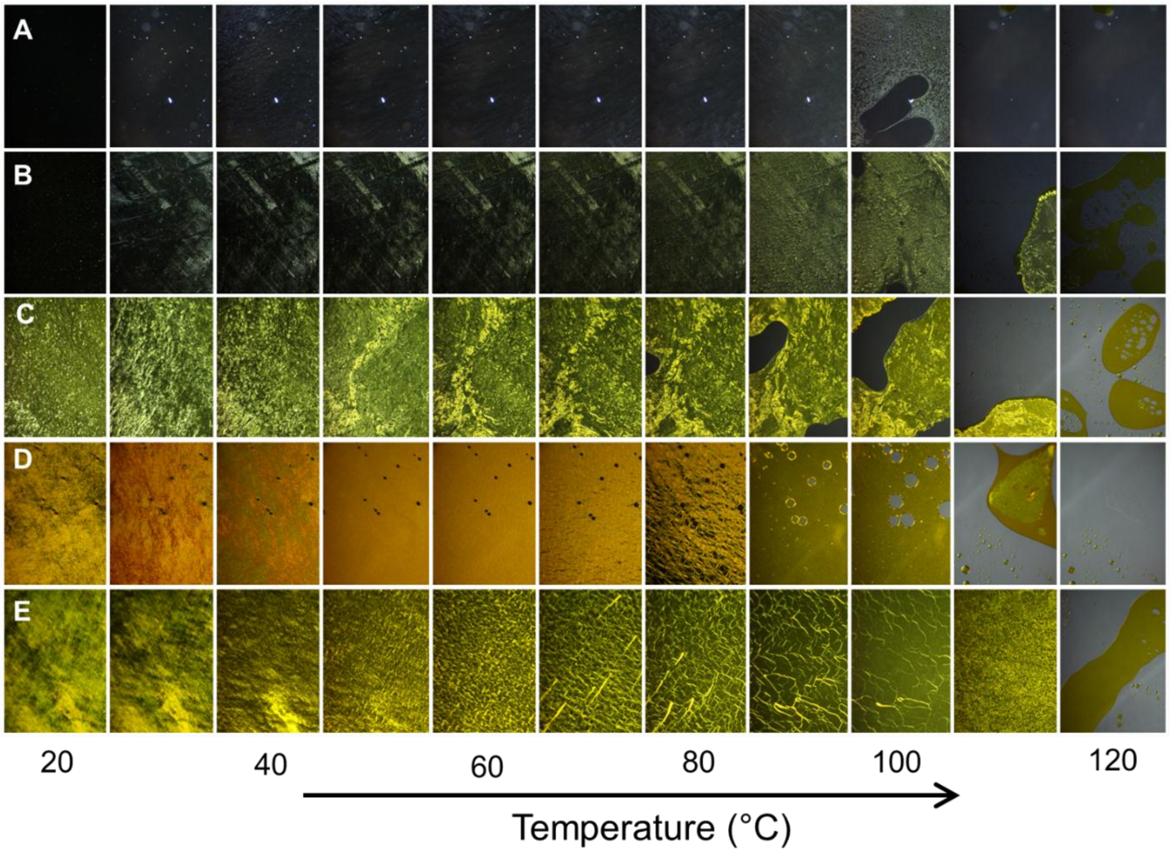

Figure S11. Polarized optical micrographs for $C_{10}AzoC_4E_4$ at increasing concentrations (wt%) in water on heating from 20 - 120°C.

6 Temperature-dependence of AzoPS-water LLCs

The temperature-dependence of $C_8AzoC_4E_4$ LLC mesophases was investigated further using SAXS with *in-situ* pseudo-DSC. For 50 wt% $C_8AzoC_4E_4$, no LLC mesophases were observed in the SAXS-DSC set-up (Figure S12a). Some crystallization was observed at high temperatures (80°C), with Bragg peaks appearing just before sample clearing and evaporation of water, which is visible as a large endothermic peak in the *in-situ* DSC thermogram (Figure S13). *Ex-situ* DSC thermograms at this concentration show phase transitions only below 20°C (Figure S14), that cannot be assigned to associated structural phase transitions.

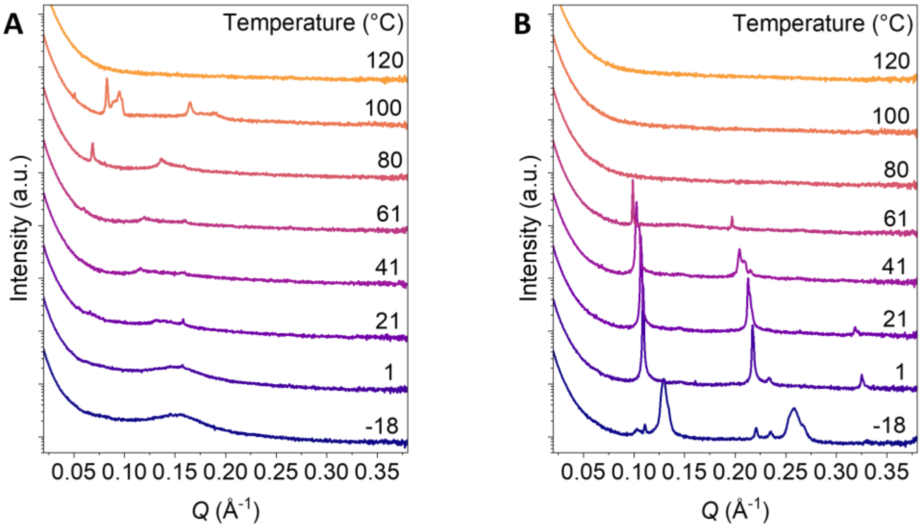

Figure S12. SAXS patterns for $C_8AzoC_4E_4$ at (a) 50 wt% and (b) 90 wt% in water, on heating from -18 to 120°C, showing the formation of Bragg diffraction peaks, characteristic of lamellar LLC phases, and their loss on heating due to clearing to form an isotropic phase.

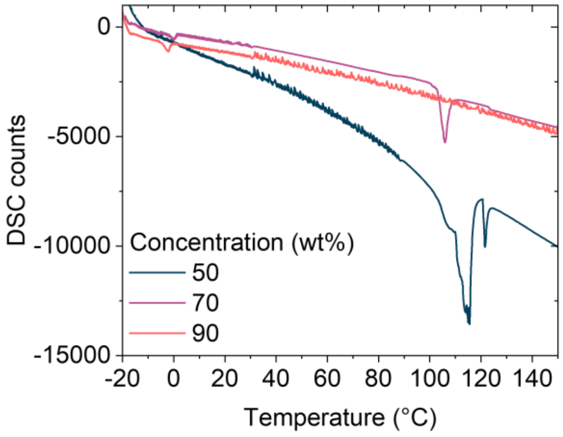

Figure S13. *In-situ pseudo*-DSC thermograms for $C_8AzoC_4E_4$ LLCs at 50 wt%, 70 wt% and 90 wt% in water on heating from -20 to 150°C.

For 70 wt%, the *d* spacing increases with increasing temperature (Table S9). Specifically, the phase transition occurring between 21 and 41°C results in a sharpening of the peaks (see main text, Figure 5), suggesting the dominance of a single lamellar phase rather that the three distinct lamellar phases present initially, which gave rise to the shoulder regions discussed previously (Figure S13). The lamellar re-ordering is visible using POM as the development of a sharper, striped pattern instead of the dense, smoke-like pattern present previously (Figure S10). From 41 to 100°C, the Bragg peaks shift to higher *Q*, corresponding to a decrease in *d* spacing from 8.8 to 7.3 nm, attributed to a dehydration of the AzoPS head groups (Table S9).

At 90 wt%, a L_c-L_α transition occurs at 0°C, as shown by the emergence of Bragg peaks at lower *Q* distances (Figure S12), corresponding to larger *d* spacings (Table S10). This is associated with an endothermic enthalpy change in the *in-situ* DSC thermogram (Figure S12). For both 70 and 90 wt%, the phase change occurs *via* the growth of a secondary set of peaks, rather than a gradual peak shift, suggesting the mechanism for this change is the heterogenous formation of the new phase, rather than a gradual swelling of the lamellar layers. Interestingly, the 90 wt% LLC clears at a lower temperature than the 70 wt% sample (60°C *cf.* 100°C), visible using both SAXS and POM (SI, Figures S10 and S12). This L_α-I_0 transition is associated with an endothermic enthalpy change in the *in-situ* DSC thermogram (Figure S13).

Table S9. Positions of the Bragg peaks in the SAXS curves for $C_8AzoC_4E_4$ (70 wt% in water) on increasing the temperature. The peaks have been sorted into those assigned to a primary and secondary lamellar phase, with the lamellar spacing (d_1 and d_2) calculated using the position of the primary peak for each phase. Above 80°C the scattering curve contains no Bragg peaks, indicating an isotropic phase.

Temperature	Peak positions - primary (Å^{-1})			Peak positions - secondary (Å^{-1})			*d* spacing (nm)	
(°C)	Q_0	Q_1	Q_2	Q_0	Q_1	Q_2	d_1	d_2
-18	0.1301	0.2594	0.3894				4.83	
-15	0.1295	0.2584	0.3874				4.85	
-11	0.0922	0.1838		0.1273	0.2540	0.3811	4.94	6.82
-5	0.1007	0.1991	0.2983	0.1245	0.2493	0.3735	5.05	6.24
1	0.0850	0.1703	0.2612				7.39	
6	0.0840	0.1659	0.2490				7.48	
11	0.0834	0.1653	0.2490				7.53	
16	0.0818	0.1631					7.68	
21	0.0293	0.0561	0.1019	0.0790	0.1612		21.5	7.95
26	0.0273	0.0546	0.0845	0.0781	0.1593		11.5	8.05
31	0.0771	0.1524					8.15	
36	0.0740	0.1471		0.0765	0.1501		8.49	8.21
41	0.0712	0.1417	0.2155				8.83	
46	0.0715	0.1421	0.2151				8.79	
51	0.0718	0.1430	0.2148				8.75	
56	0.07214	0.1439	0.2161				8.67	
60	0.0727	0.1452	0.2170				8.64	
65	0.0734	0.1465	0.2195				8.56	
70	0.0740	0.1474	0.2224				8.49	
75	0.0749	0.1490					8.38	
80	0.0756	0.1502					8.32	
85	0.0762	0.1518					8.25	
90	0.0778	0.1552					8.08	
95	0.0818	0.1637					7.68	
100	0.0847	0.1693					7.42	
105 - 145				Isotropic				

Table S10. Positions of the Bragg peaks in the SAXS curves for $C_8AzoC_4E_4$ (90 wt% in water) on increasing the temperature. The peaks have been sorted into those assigned to a primary and secondary lamellar phase, with the lamellar spacing (d_1 and d_2) calculated using the position of the primary peak for each phase. Above 80°C the scattering curve contains no Bragg peaks, indicating an isotropic phase.

Temperature	Peak positions - primary (Å^{-1})			Peak positions - secondary (Å^{-1})			*d* spacing (nm)	
(°C)	Q_0	Q_1	Q_2	Q_0	Q_1	Q_2	d_1	d_2
-18	0.1107	0.2205		0.1295	0.2578		5.68	4.85
-15	0.1107	0.2205		0.1292	0.2575		5.68	4.86
-10	0.1104	0.2202	0.3293	0.1289	0.2569		5.69	4.88
-5	0.1104	0.2202	0.3293	0.1279	0.2553		5.69	4.91
1	0.1088	0.2173	0.3252				5.77	
6	0.1085	0.2161	0.3237				5.79	
11	0.1079	0.2151	0.3221				5.82	
16	0.1072	0.2139	0.3202				5.86	
21	0.1066	0.2126	0.3186				5.89	
26	0.1060	0.2114	0.3164				5.93	
31	0.1051	0.2095	0.3136				5.98	
36	0.1038	0.2073		0.1052	0.2112		6.05	5.97
41	0.1025	0.2045		0.1047	0.2086		6.13	6.00
46	0.0972	0.1938		0.0997	0.1991		6.46	6.30
51	0.0975	0.1944		0.1022	0.2048		6.44	6.15
56	0.0982	0.1957					6.40	
61	0.0988	0.1969					6.36	
66 - 145				Isotropic				

Ex-situ DSC measurements were used to obtain quantitative values for the enthalpy changes associated with phase transitions in the AzoPS LLCs (Figure S14). Three separate scans were taken on identical samples and the results averaged, with errors given by the standard deviation (Table S11).

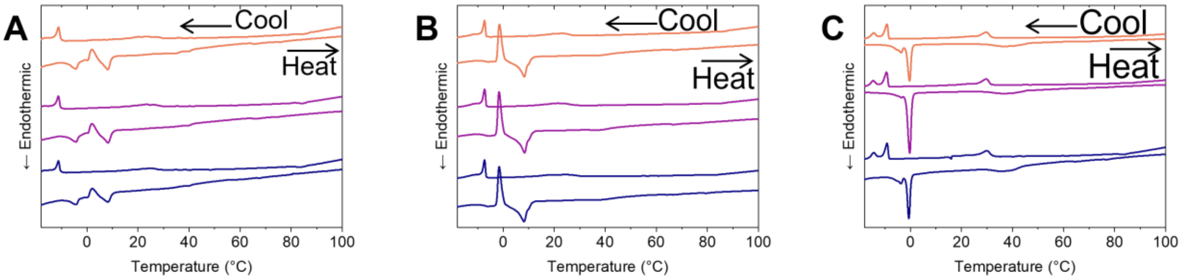

Figure S14. DSC thermograms for three separate scans of identical $C_8AzoC_4E_4$ LLC samples at (a) 50 wt%, (b) 70 wt% and (c) 90 wt% in water on heating and cooling.

Table S11. Summary of results from integration of peaks in *ex-situ* DSC thermograms of $C_8AzoC_4E_4$ LLCs at increasing concentrations in water. The upper and lower temperature limits of integration (°C) and enthalpy changes (Δ*H*) are given for heating and cooling, as an average over three samples, unless indicated otherwise. Errors are calculated from the standard deviation of these results.

Concentration (wt%)	Cycle	Lower limit (°C)	Upper limit (°C)	Δ*H* (J g^{-1})	Δ*H* (kJ mol^{-1})
50		-11	0	3.2 ± 0.5	0.9 ± 0.2
	Heat 1	0	5	-2.9 ± 0.3	-0.8 ± 0.1
		5	14	3.5 ± 1.9	1.0 ± 0.5
		29†	44†	1.5†	0.4†
	Cool	-16	-9	-2.4 ± 0.1	-0.70 ± 0.03
		11	32	-2.9 ± 0.5	-0.8 ± 0.1
70		-4	2	11.2 ± 0.4	4.4 ± 0.2
	Heat 1	2	13	-12.1 ± 2.1	-4.8 ± 0.8
		27	51	2.6 ± 0.5	1.0 ± 0.2
	Cool	-17	-4	-5.9 ± 1.1	-2.3 ± 0.4
		7	34	-4.3 ± 0.3	-1.7 ± 0.1
90	Heat 1	-10	5	21.3 ± 4.0	10.7 ± 2.0
		24	48	8.2 ± 1.4	4.2 ± 0.7
	Cool	-19	-7	-9.8 ± 0.3	-4.9 ± 0.1
		17	34	-6.2 ± 1.3	-3.1 ± 0.7

† Peak only present in one of three runs.

7 Characterization of AzoPS-ethylene glycol LLCs

Formation of LLC mesophases using ethylene glycol as the solvent, instead of water, was investigated using SAXS and POM. Above 50 wt% in ethylene glycol, $C_{10}AzoC_4E_4$ forms L_α LLC mesophases, as evidenced by the formation of Bragg peaks in a Q ratio of 1:2:3 in SAXS (Figure S15) and birefringent, smoke-like textures under POM (Figure S16). As in AzoPS-water LLCs, the d spacing of the L_α phase decreases on increasing $C_{10}AzoC_4E_4$ concentration (from 13.7 to 7.1 nm, at 50 and 90 wt%). $C_8AzoC_4E_4$ also forms LLC mesophases at concentrations of 50 – 90 wt% in ethylene glycol, as seen by birefringent patterns under POM (Figure S17). These are likely to be L_α mesophases but cannot be assigned unequivocally without SAXS confirmation.

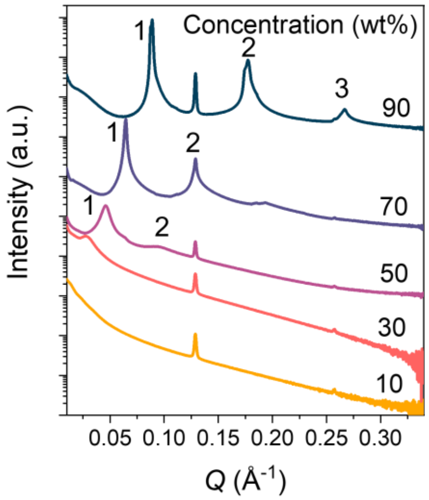

Figure S15. SAXS plots for $C_{10}AzoC_4E_4$, in the E isomer for increasing concentrations (wt%) in ethylene glycol. Bragg diffraction peaks at 50 wt% and above are in the characteristic ratio of 1:2:3, indicating the presence of lamellar LLC phases.

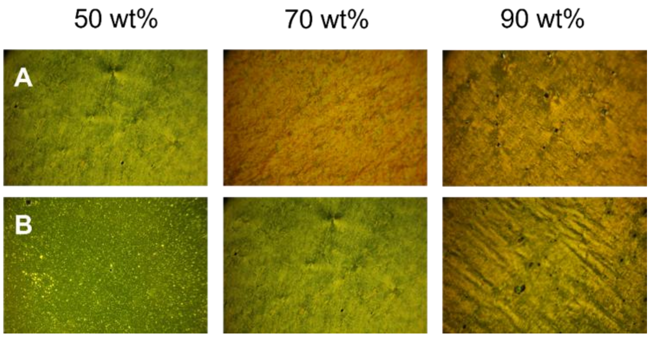

Figure S16. Polarized optical micrographs for (a) $C_8AzoC_4E_4$ and (b) $C_{10}AzoC_4E_4$ in the E isomer at increasing concentrations (wt%) in ethylene glycol at 20°C showing the formation of birefringent, stripey and smoke-like textures characteristic of LLC mesophases.

8 Isomerization Studies

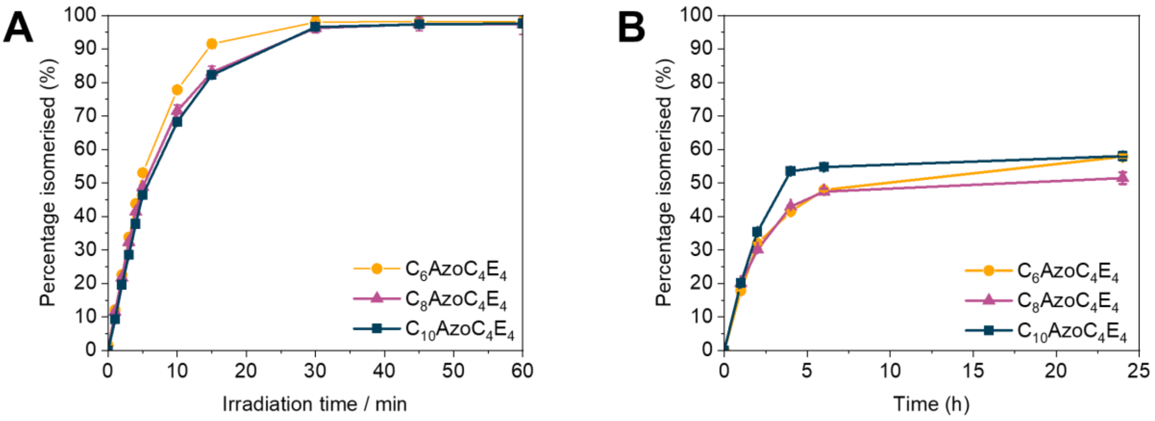

Figure S17. Percentage isomerized for AzoPS of increasing alkyl tail length ($C_6AzoC_4E_4$, $C_8AzoC_4E_4$, and $C_{10}AzoC_4E_4$) with increasing UV (365 nm) irradiation time, as determined using NMR spectroscopy, for AzoPS (a) in solution (10 mM in DMSO-d_6) and (b) at 100 wt%.

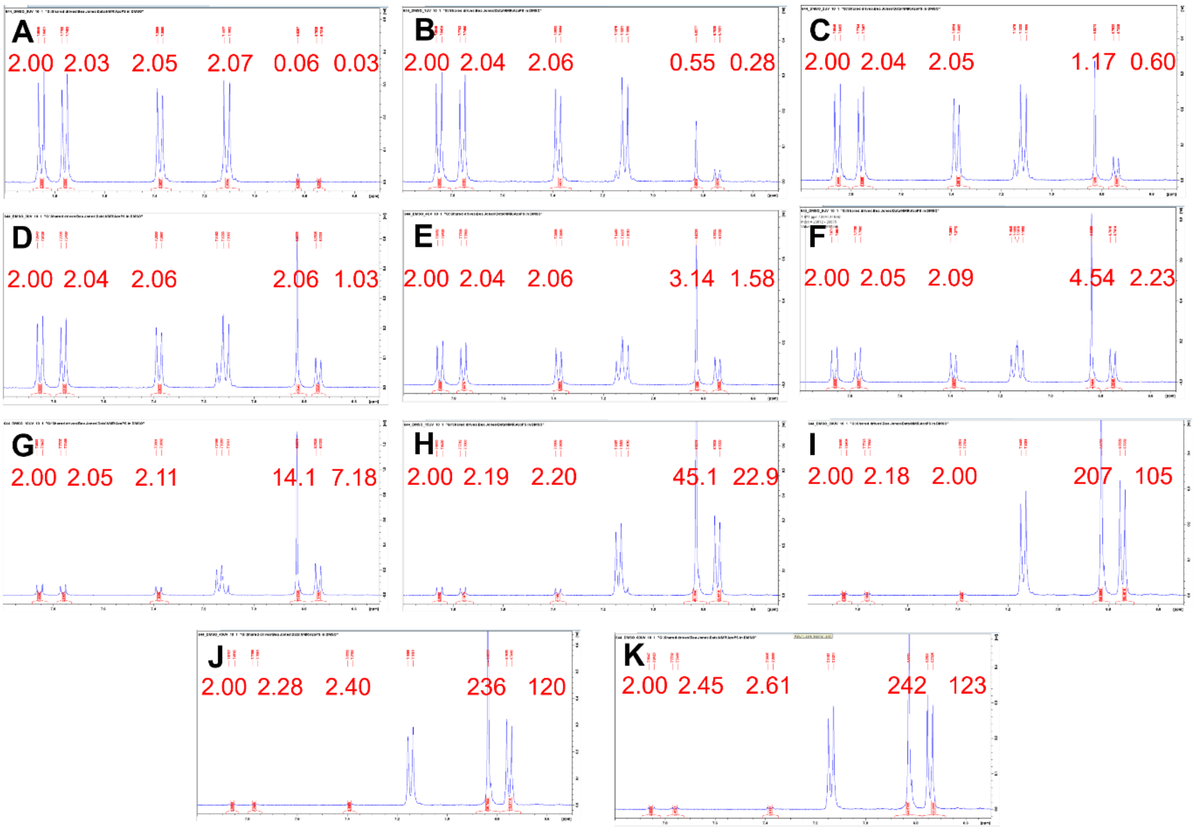

Figure S18. ^{1}H NMR spectra for $C_6AzoC_4E_4$ in d_6-DMSO (10 mM) after (a) 0 min, (b) 1 min, (c) 2 min, (d) 3 min, (e) 4 min, (f) 5 min, (g) 10 min, (h) 15 min, (i) 30 min, (j) 45 min, and (k) 60 min of UV (365 nm) irradiation. The integrals used to calculate the percentage isomerized have been enlarged.

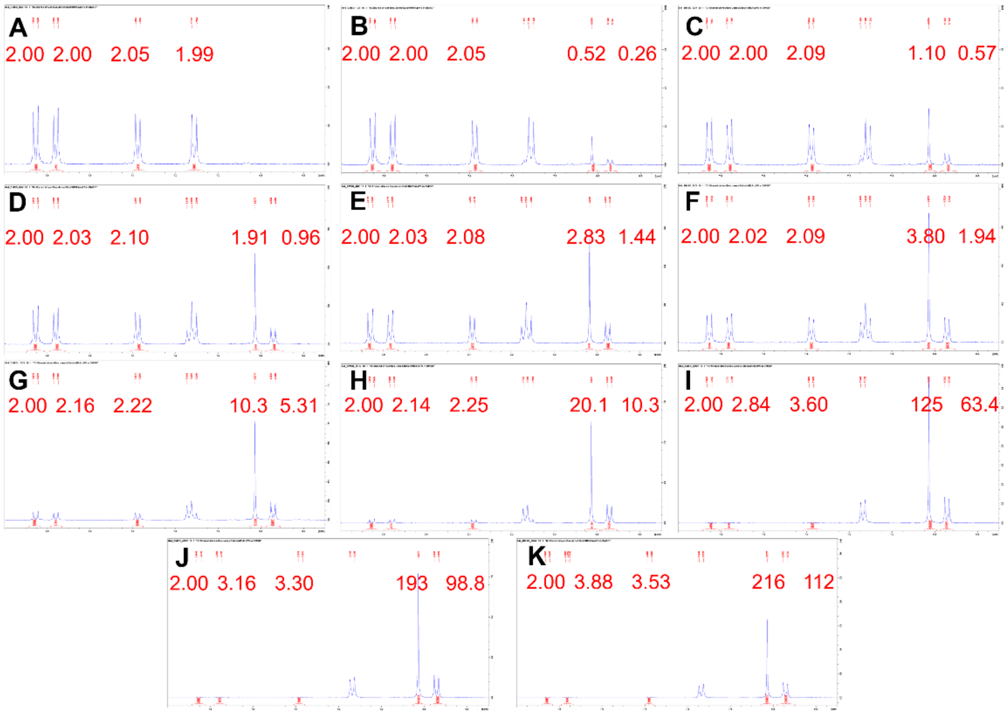

Figure S19. ^{1}H NMR spectra for $C_8AzoC_4E_4$ in d_6-DMSO (10 mM) after (a) 0 min, (b) 1 min, (c) 2 min, (d) 3 min, (e) 4 min, (f) 5 min, (g) 10 min, (h) 15 min, (i) 30 min, (j) 45 min, and (k) 60 min of UV (365 nm) irradiation. The integrals used to calculate the percentage isomerized have been enlarged.

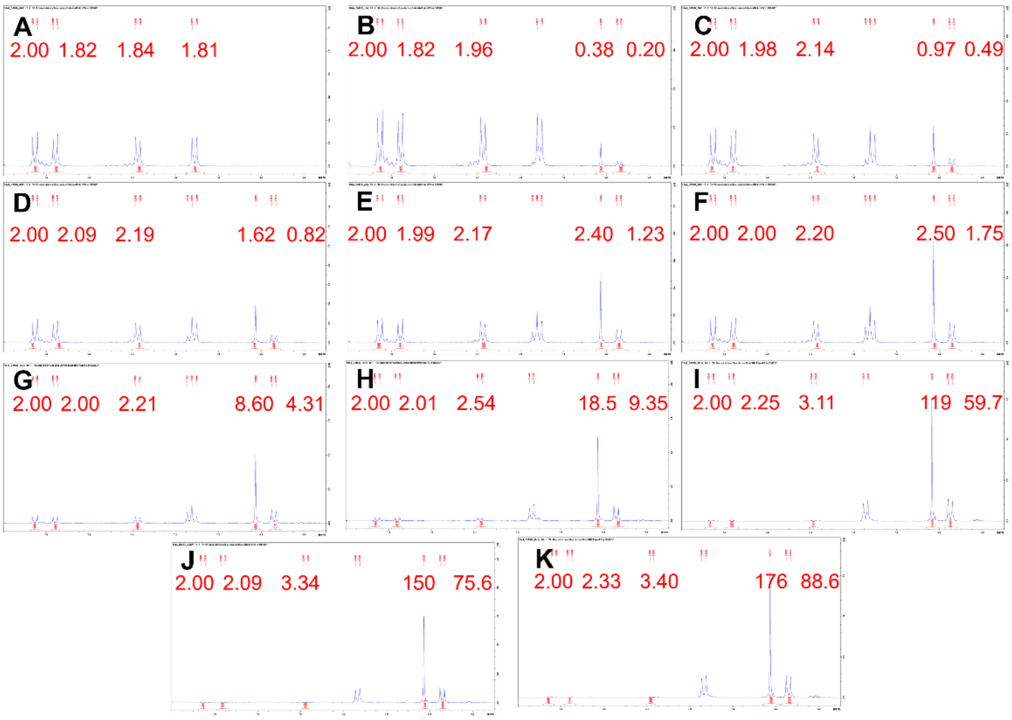

Figure S20. ^{1}H NMR spectra for $C_{10}AzoC_4E_4$ in d_6-DMSO (10 mM) after (a) 0 min, (b) 1 min, (c) 2 min, (d) 3 min, (e) 4 min, (f) 5 min, (g) 10 min, (h) 15 min, (i) 30 min, (j) 45 min, and (k) 60 min of UV (365 nm) irradiation. The integrals used to calculate the percentage isomerized have been enlarged.

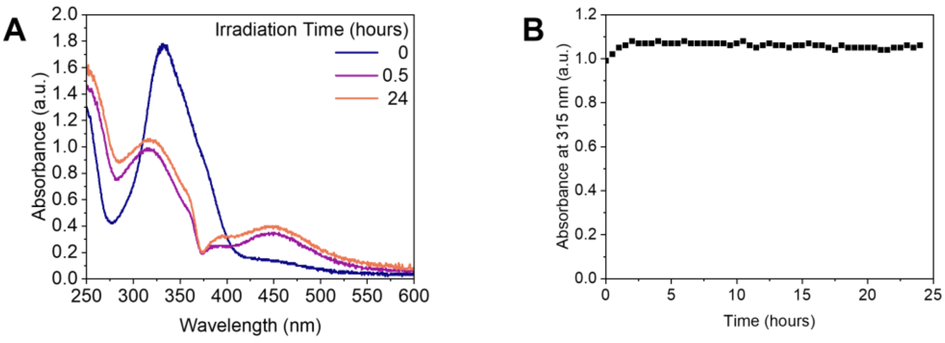

Figure S21. (a) UV-Vis absorbance spectra for $C_8AzoC_4E_4$ (100 μM in water) before UV irradiation, after 0.5 and 24 hours of UV irradiation. (b) Plot of the absorbance at in the *Z*-rich PSS over time, with constant UV (365 nm) irradiation.

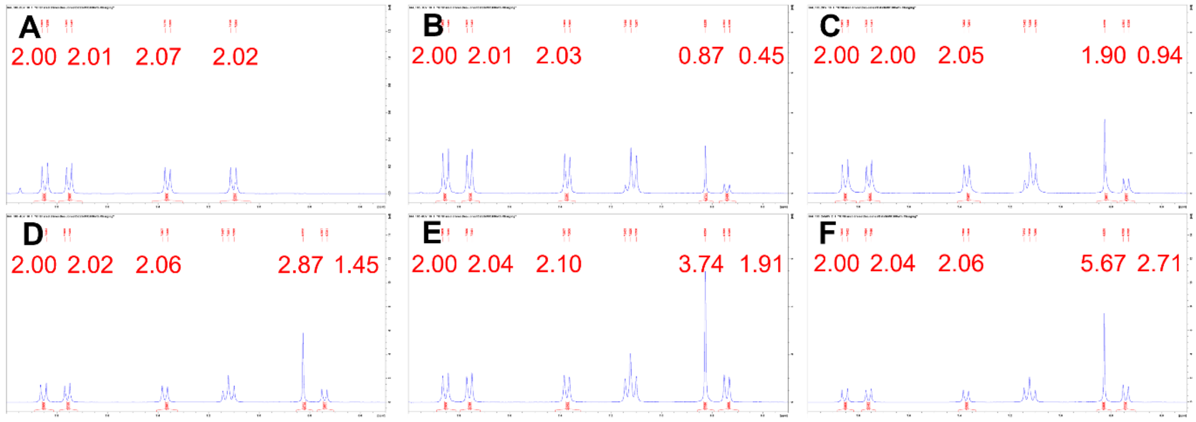

Figure S22. ^{1}H NMR spectra for $C_6AzoC_4E_4$ in d_6-DMSO after (a) 0 min, (b) 1 h, (c) 2 h, (d) 4 h, (e) 6 h, (f) 24 h UV (365 nm) irradiation in the thermotropic LC state (100 wt%). The integrals used to calculate the percentage isomerized have been enlarged.

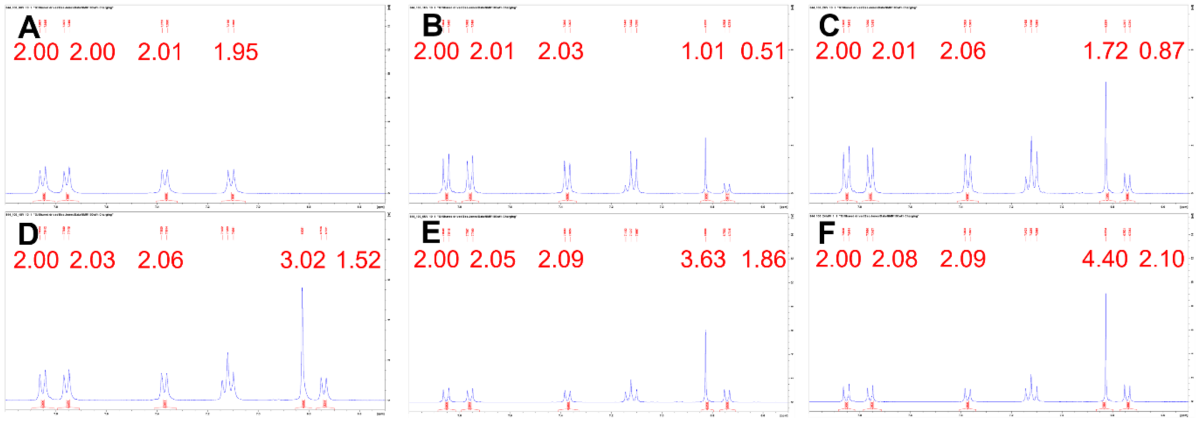

Figure S23. ^{1}H NMR spectra for $C_8AzoC_4E_4$ in d_6-DMSO after (a) 0 min, (b) 1 h, (c) 2 h, (d) 4 h, (e) 6 h, (f) 24 h UV (365 nm) irradiation in the thermotropic LC state (100 wt%). The integrals used to calculate the percentage isomerized have been enlarged.

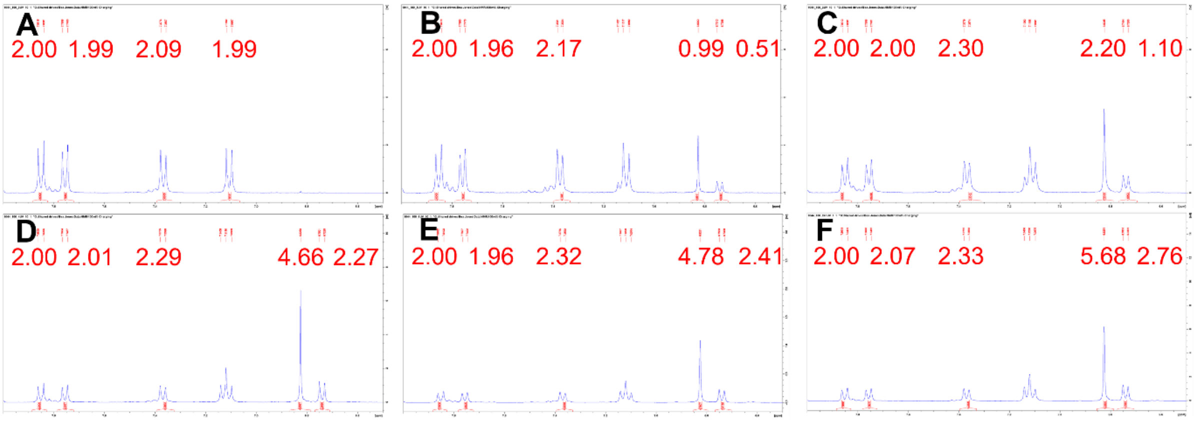

Figure S24. ^{1}H NMR spectra for $C_{10}AzoC_4E_4$ in d_6-DMSO after (a) 0 min, (b) 1 h, (c) 2 h, (d) 4 h, (e) 6 h, (f) 24 h UV (365 nm) irradiation in the thermotropic LC state (100 wt%). The integrals used to calculate the percentage isomerized have been enlarged.

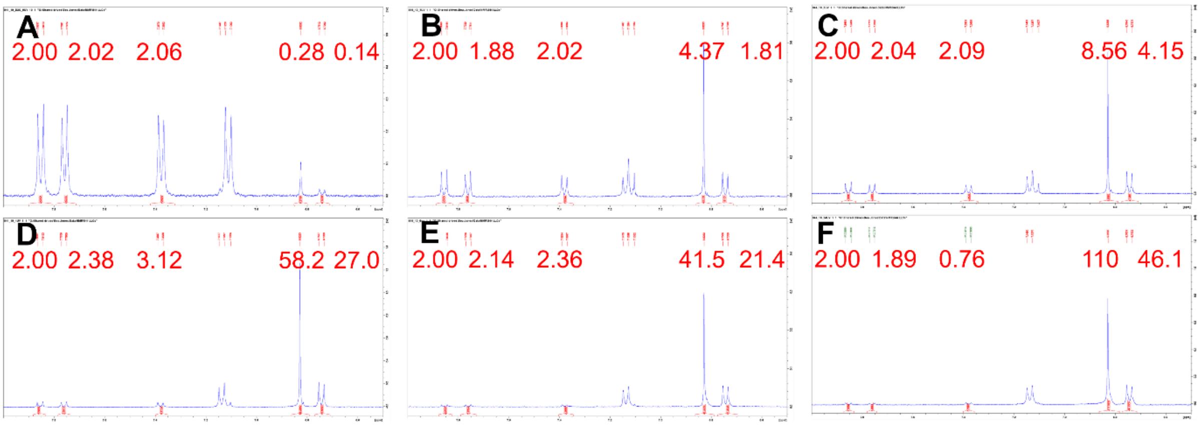

Figure S25. ^{1}H NMR spectra for $C_8AzoC_4E_4$ in d_6-DMSO after (a) 0 min, (b) 1 h, (c) 2 h,(d) 4 h, (e) 6 h, (f) 24 h UV (365 nm) irradiation at a concentration of 10 wt% in D_2O. The integrals used to calculate the percentage isomerized have been enlarged.

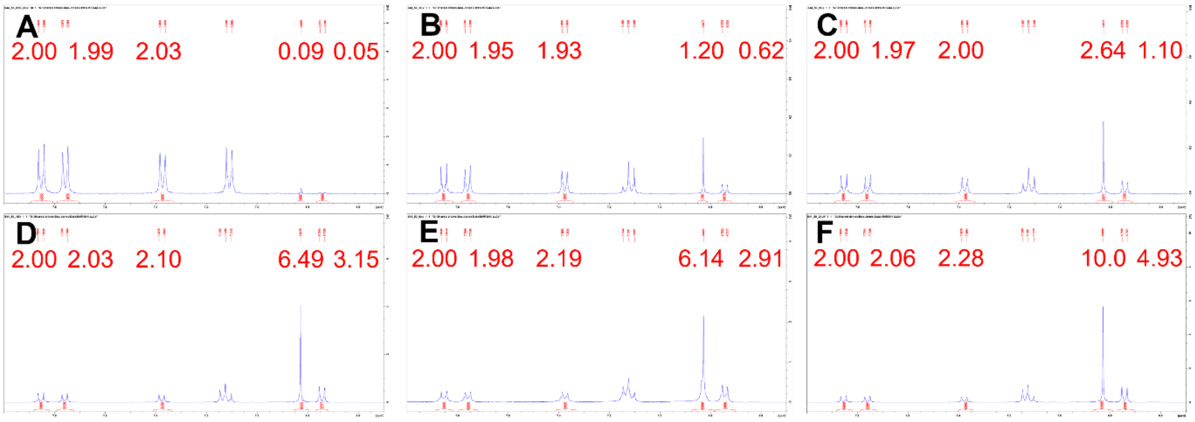

Figure S26. ^{1}H NMR spectra for $C_8AzoC_4E_4$ in d_6-DMSO after (a) 0 min, (b) 1 h, (c) 2 h,(d) 4 h, (e) 6 h, (f) 24 h UV (365 nm) irradiation at a concentration of 50 wt% in D_2O, in the swollen lamellar LLC phase. The integrals used to calculate the percentage isomerized have been enlarged.

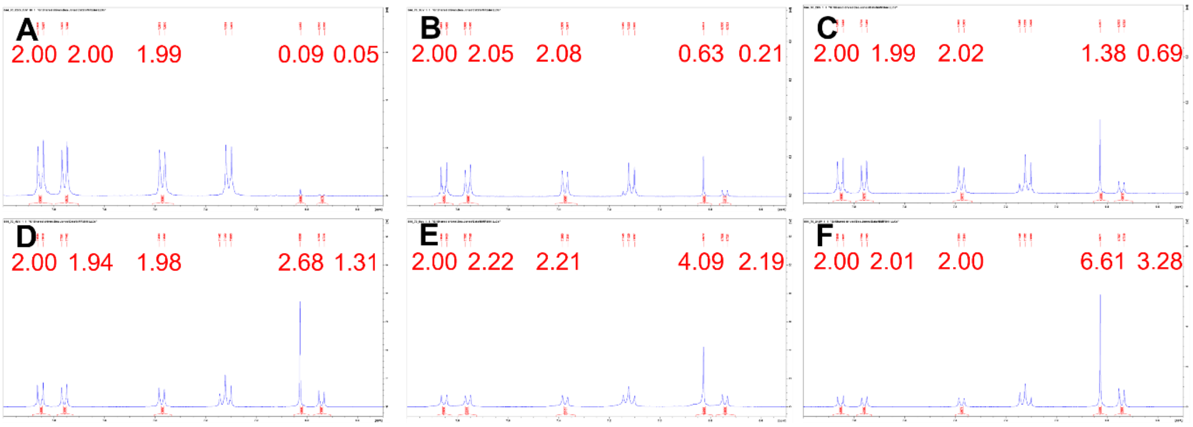

Figure S27. ^{1}H NMR spectra for $C_8AzoC_4E_4$ in d_6-DMSO after (a) 0 min, (b) 1 h, (c) 2 h,(d) 4 h, (e) 6 h, (f) 24 h UV (365 nm) irradiation at a concentration of 70 wt% in D_2O, in the tightly-packed lamellar LLC phase. The integrals used to calculate the percentage isomerized have been enlarged.

9 Characterization of AzoPS-D_2O LLCs

The effect of switching the solvent from H_2O to D_2O on LLC formation was investigated using SAXS and POM. This was to ensure that NMR studies using D_2O as a solvent are valid to compare with the H_2O system used for the rest of the experiments in this study. With D_2O as a solvent, an isotropic micellar (I_0) phase formed from 10 – 30 wt% and lamellar (L_α) phase from 50 – 90wt%, as observed in H_2O (Figures S27 and S28). For the purposes of this study, as the same LLC phases formed with H_2O and D_2O, the degree of isomerization within AzoPS-D_2O LLC phases was used as a reasonable approximation to the corresponding isomerization degrees in AzoPS-H_2O LLCs.

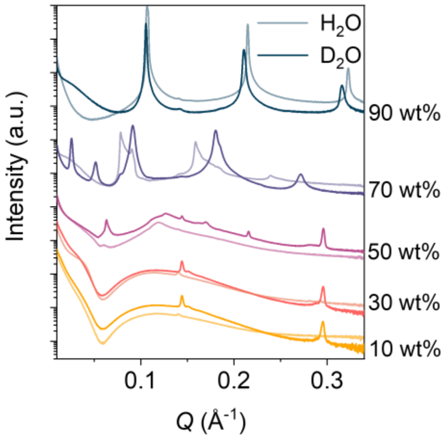

Figure S28. SAXS patterns indicating the formation of LLC phases on increasing concentration (wt%) of $C_8AzoC_4E_4$ in the *E* isomer in H_2O and D_2O. Broad interaction humps indicate the formation of isotropic phases for concentrations of 10 – 30 wt%. Bragg diffraction peaks are characteristic of a lamellar LLC phase for concentrations greater than 50 wt%. Note that there the peak positions are shifted in D_2O in comparison to H_2O, indicating a change in the *d* spacing of the lamellar phase.

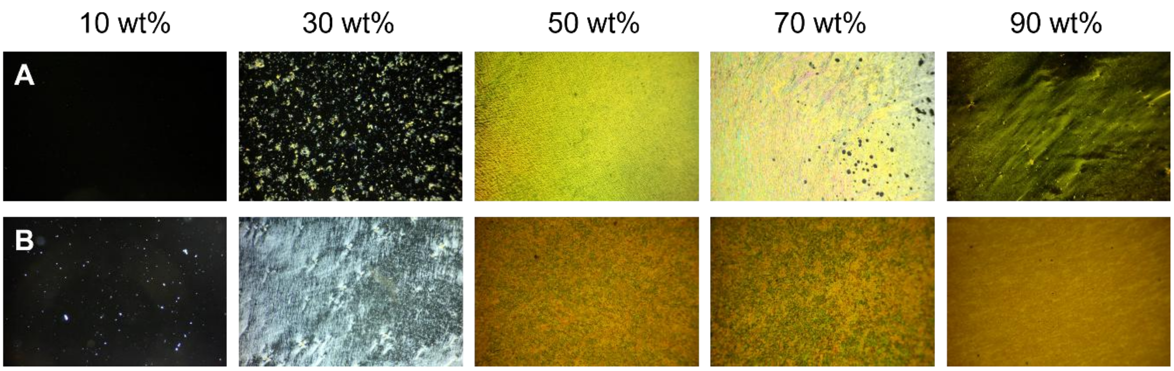

Figure S29. POM micrographs for LLCs formed from $C_8AzoC_4E_4$ in the *E* isomer at increasing concentrations (wt%) in (a) D_2O and (b) H_2O at 20°C.

10 Determination of AzoPS thermal half-life

To determine the thermal half-life of the *Z* isomer for each of the AzoPS structures, time-dependent UV-vis absorbance spectra were taken at 45, 50, 55, 60 and 65°C (Figure S30). The absorbance at the position of the absorbance maximum peak in the *E* isomer (~330 nm but this varied slightly with AzoPS sample, Figures S30-32) was then used to calculate the percentage in the *Z* isomer (%*Z*), as described in the Methods section (Equation 1). Plots of ln(%*Z*) against time were used to fit the first-order rate parameter (*k*) at each temperature, which were used in the Eyring plots (Figure S34). The thermodynamic parameters were calculated using the Eyring equation (Equation 3) and thermal half-life at 20°C calculated by extrapolating back the straight line (Table S12).

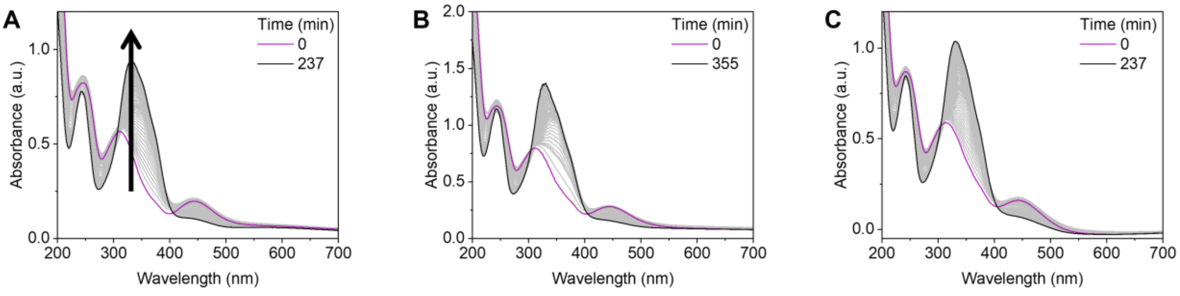

Figure S30. Representative UV-vis absorbance spectra for the thermal reversion of AzoPS at 45°C, showing the transition from the *Z*-PSS (time = 0 min) to the *E* state (time = 237 min) *via* the series of grey spectra, as measured every 3 minutes, for (a) $C_6AzoC_4E_4$, (b) $C_8AzoC_4E_4$ and (c) $C_{10}AzoC_4E_4$.

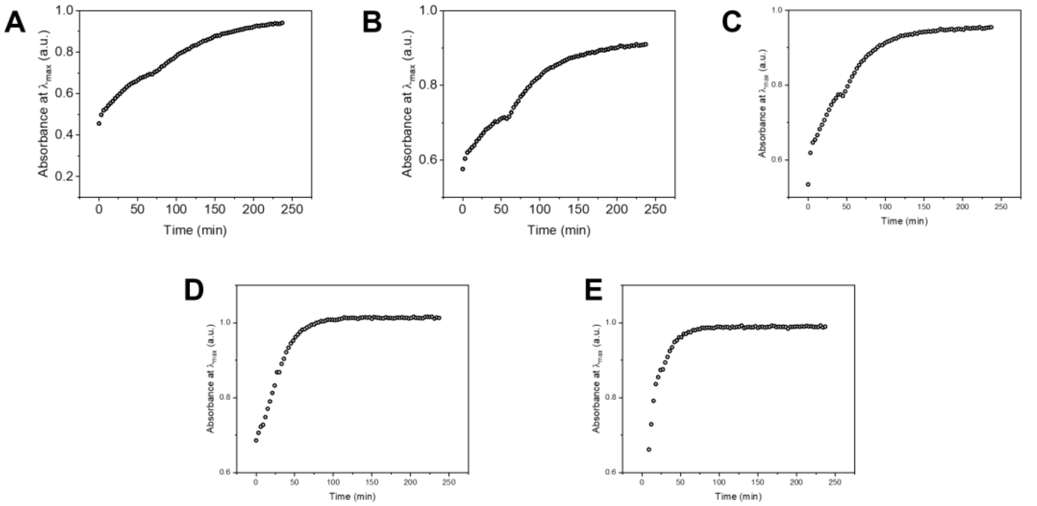

Figure S31. Plots of absorbance of $C_6AzoC_4E_4$ at the *E* λ_{max} (~330 nm but varied for each temperature) with increasing time at (a) 45, (b) 50, (c) 55, (d) 60 and (e) 65°C.

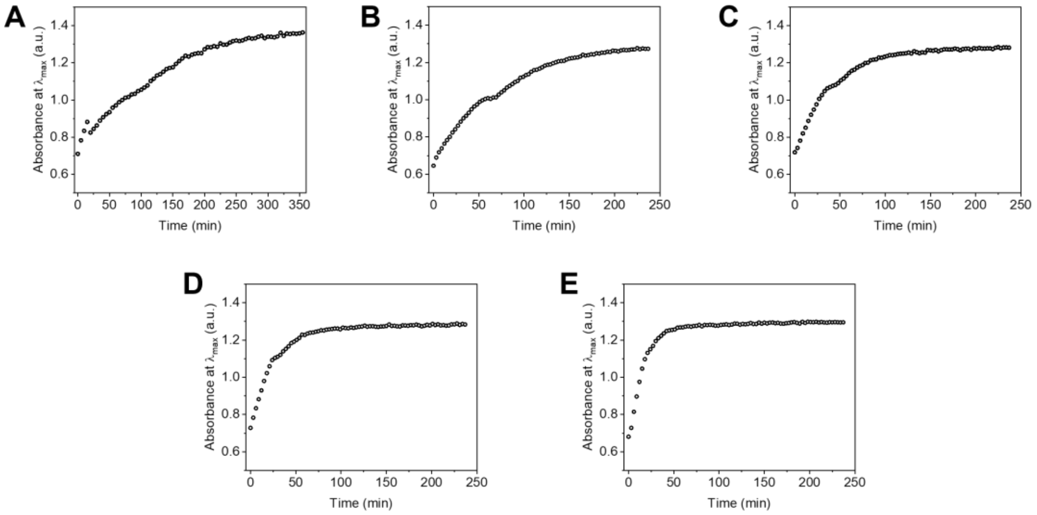

Figure S32. Plots of absorbance of $C_8AzoC_4E_4$ at the *E* λ_{max} (~330 nm but varied for each temperature) with increasing time at (a) 45, (b) 50, (c) 55, (d) 60 and (e) 65°C.

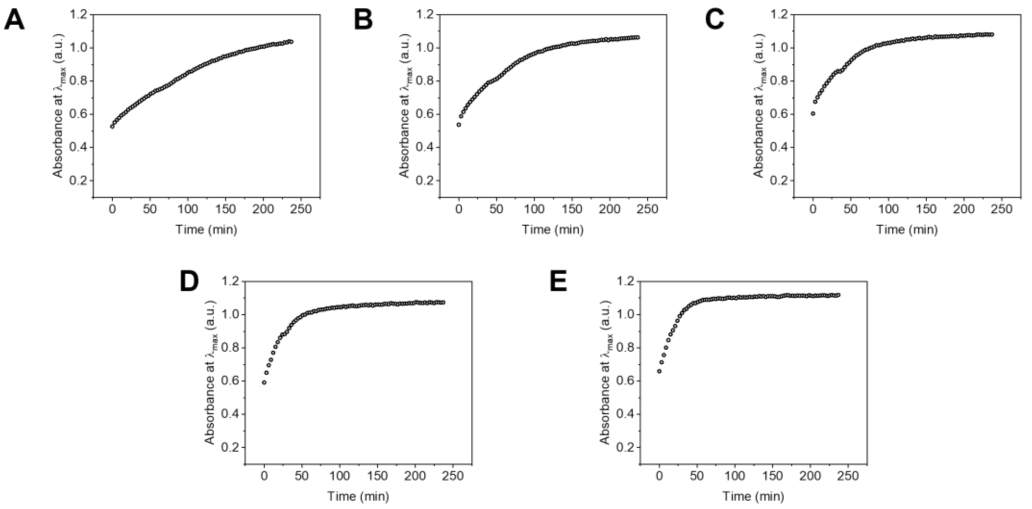

Figure S33. Plots of absorbance of $C_{10}AzoC_4E_4$ at the *E* λ_{max} (~330 nm but varied for each temperature) with increasing time at (a) 45, (b) 50, (c) 55, (d) 60 and (e) 65°C.

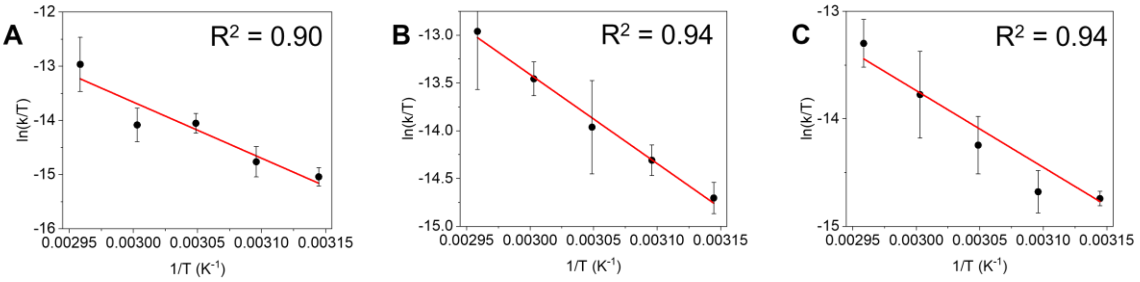

Figure S34. Eyring plots for the thermal stability of (a) $C_6AzoC_4E_4$, (b) $C_8AzoC_4E_4$ and (c) $C_{10}AzoC_4E_4$. Error bars for each temperature are given from the standard error in the straight-line fit used to obtain the value of the first-order rate parameter, *k*, at this temperature.

Table S12. Summary of the thermodynamic parameters for the thermal relaxation of *Z* AzoPS. Enthalpy change (ΔH), entropy change (ΔS) and thermal half-life ($t_{1/2}$) at 20°C.

AzoPS	ΔH / kJ mol^{-1}	ΔS / J K^{-1} mol^{-1}	$t_{1/2}$ / h
$C_6AzoC_4E_4$	-86 ± 17	1.2 ± 0.4	228 ± 10
$C_8AzoC_4E_4$	-77 ± 4	1.2 ± 0.1	116 ± 1.3
$C_{10}AzoC_4E_4$	-59 ± 9	1.2 ± 0.5	65 ± 1.5

11 SAXS with *in-situ* DSC for UV-irradiated AzoPS LCs

For $C_8AzoC_4E_4$ in thermotropic LC mesophases, on UV irradiation a smectic LC phase forms with a shorter *d* spacing, due to the shorter tail-length of AzoPS with azobenzene in the bent, *Z* isomer (as discussed in the main text). In addition to this, after UV irradiation between 20 minutes and 4 hours, a new smectic phase was observed at -18°C, shown by peaks forming at lower *Q* values than the original phase for the *E* isomer (Figure S35, peaks 1* and 2*). This corresponds to a larger *d* spacing (6.7 *cf.* 5.7 nm in the *E* isomer). The larger spacing could be due to an increase in the free volume of lamellar packing, induced by the formation of a mixed-photostationary state on irradiation and resulting in a disordering of the tail groups in the amphiphile bilayer. The formation of less-ordered packing in the smectic phase formed on irradiation is shown by broader peaks in the SAXS curves, which indicates that the order in this phase is less well-defined. This disordered smectic phase melts at ~0°C, accompanied by a small endothermic peak in the *in-situ* DSC thermogram (Figure S37).

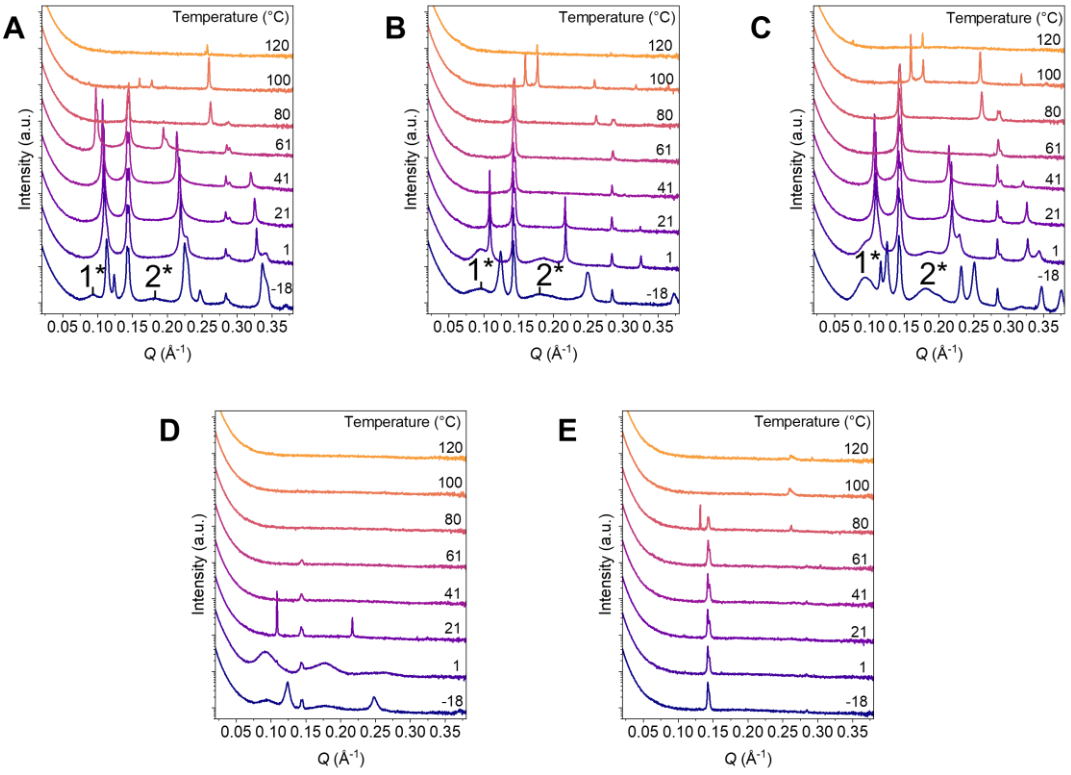

Figure S35. Variation in thermotropic liquid crystal formation for $C_8AzoC_4E_4$ on heating from -18 to 120°C with increasing UV irradiation time (a) 20 minutes, (b) 60 min, (c) 2 h, (d) 4 h, (e) 11 h.

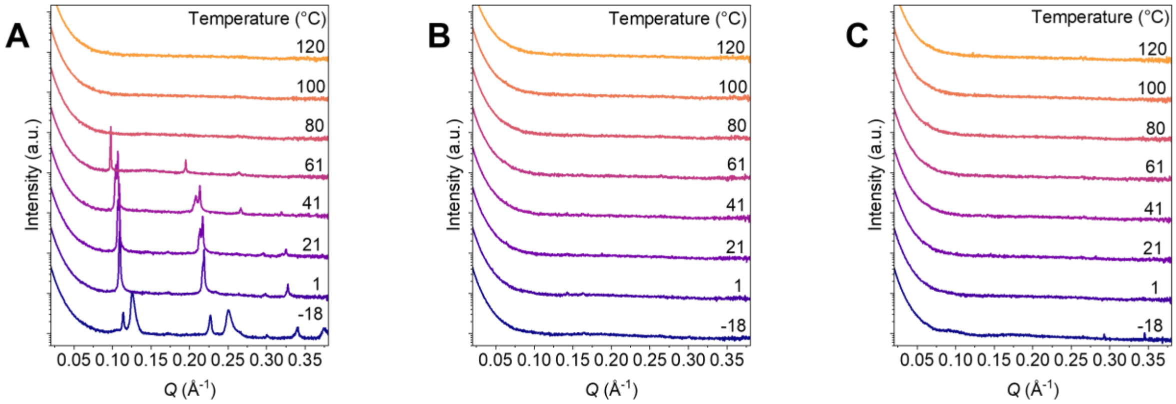

Figure S36. Variation in lyotropic liquid crystal formation for $C_8AzoC_4E_4$ (70 wt%) on heating from -18 to 120°C with increasing UV irradiation time (a) 1 h, (b) 4 h, (c) 11 h.

12 DSC Thermograms for UV-Irradiated AzoPS

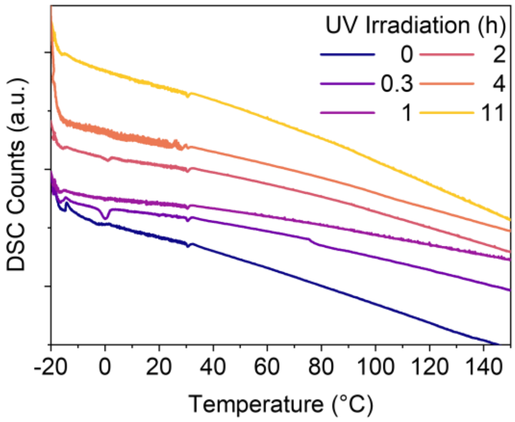

Figure S37. *In-situ pseudo*-DSC thermograms from SAXS study for $C_8AzoC_4E_4$ (100 wt%) after increasing UV irradiation times on heating from -20 to 150°C.

To determine the isomerization enthalpy for the AzoPS molecules experimentally, *ex-situ* DSC measurements were taken for AzoPS in the *Z* isomer. An exothermic, isomerization peak was measured for all AzoPS (Figure S38 and main text, Figure 7a) at ~80°C. This peak is not present on the second heating cycle, indicating that full *Z-E* isomerization occurs during the first heating ramp. The isomerization change before and after DSC scans was determined using NMR spectroscopy, giving values between 71 and 97%, which were highly sample dependent (Table S13). These values were used to calculate the theoretical isomerization enthalpy associated with 100% *Z-E* conversion for the AzoPS.

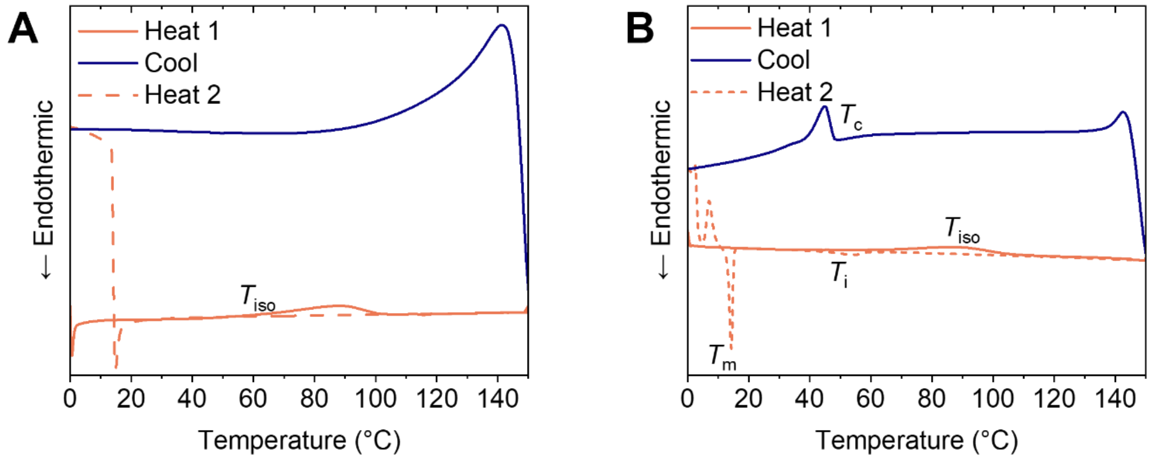

Figure S38. DSC thermograms for (a) $C_6AzoC_4E_4$ and (b) $C_{10}AzoC_4E_4$ in the *Z* isomer (irradiated in $CDCl_3$, and dried before measurement) on heating from 0 – 150°C, cooling and second heating. Labelled temperatures refer to isomerization (T_{iso}), crystallization (T_c), melting (T_m) and clearing (T_i) temperatures.

Table S13. Change in the isomerization (%) before and after DSC scans on AzoPS of increasing alkyl tail length in the *Z*-PSS, as determined using ^{1}H NMR spectroscopy in $CDCl_3$. The measured isomerization enthalpy for the 3 runs of each AzoPS sample has been given and the theoretical isomerization enthalpy, for 100% conversion between the photoisomers. The error given in the theoretical enthalpy is calculated from the standard deviation in the integration values for the characteristic *E* and *Z* peaks in the NMR spectra.

AzoPS	Run	Isomerization Change / %	Isomerization Enthalpy / J g^{-1}	
			Measured	Theoretical
$C_6AzoC_4E_4$	1	84	83	99 ± 3
	2	82	67	82 ± 1
	3	71	58	81 ± 1
	Average	79 ± 7	69 ± 12	87 ± 3
$C_8AzoC_4E_4$	1	98	88	90 ± 3
	2	76	94	124 ± 5
	3	90	86	96 ± 3
	Average	88 ± 11	89 ± 4	103 ± 7
$C_{10}AzoC_4E_4$	1	97	51	52 ± 3
	2	97	56	58 ± 4
	3	97	56	58 ± 4
	Average	96 ± 0	54 ± 3	56 ± 6

13 Density Functional Theory

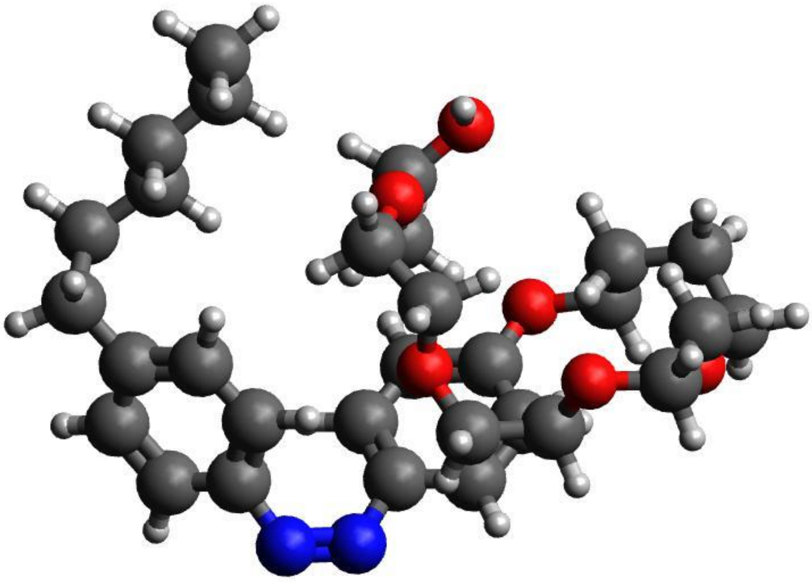

Figure S39. DFT-optimized structure of the *Z*-isomer of $C_6AzoC_4E_4$.

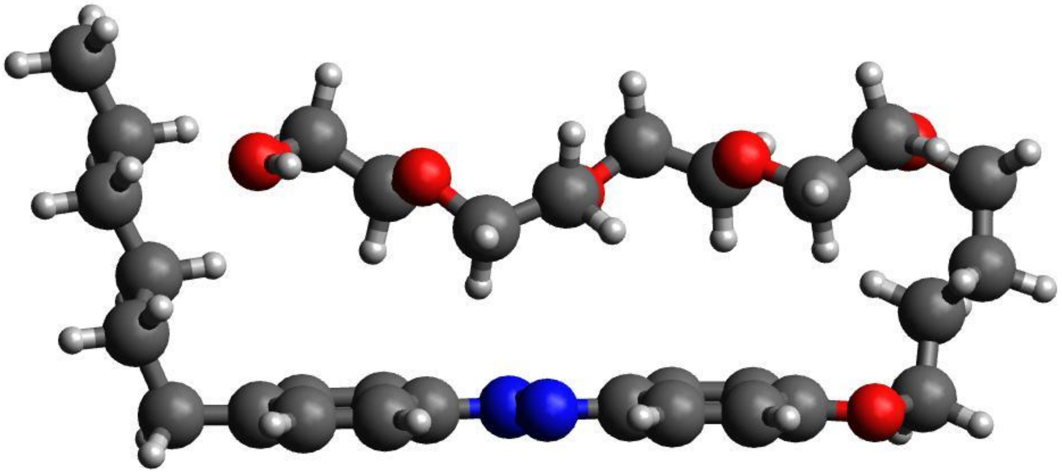

Figure S40. DFT-optimized structure of the *E*-isomer of $C_6AzoC_4E_4$.

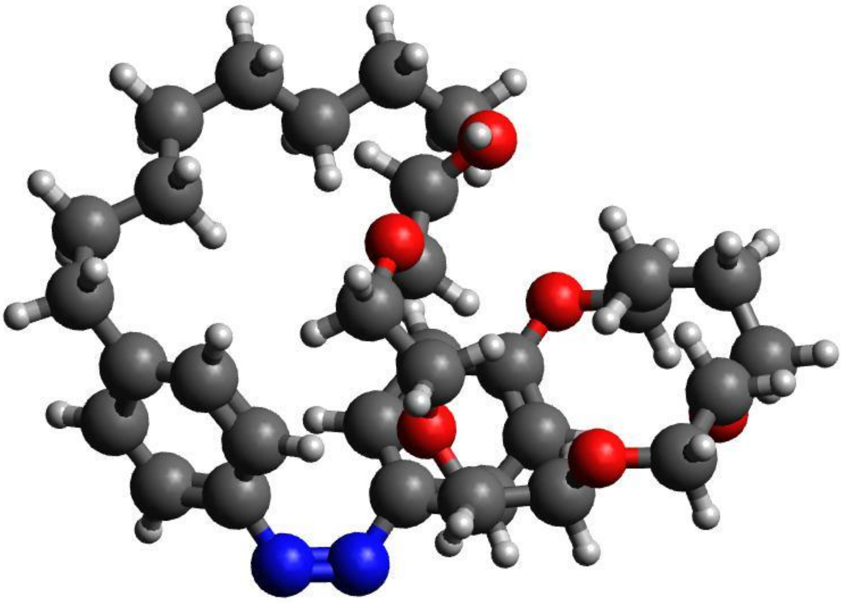

Figure S41. DFT-optimized structure of the *Z*-isomer of $C_8AzoC_4E_4$.

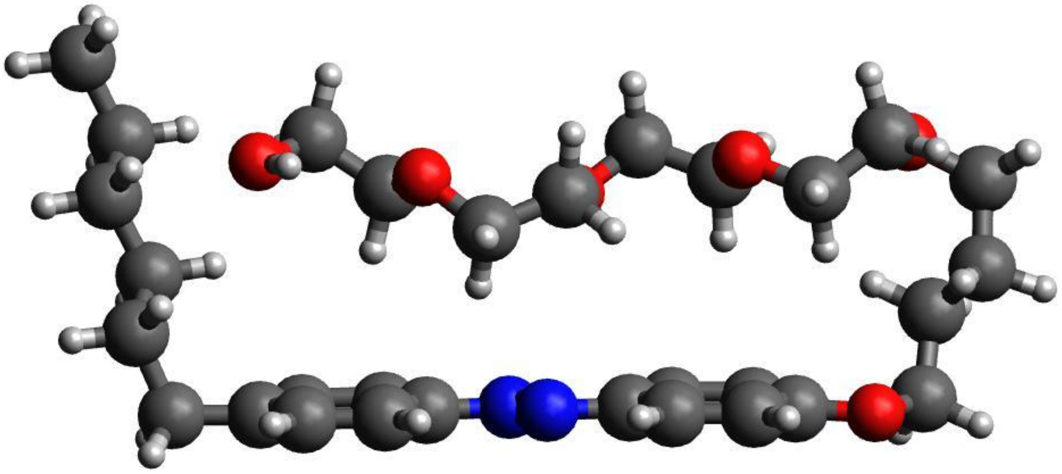

Figure S42. DFT-optimized structure of the *E*-isomer of $C_8AzoC_4E_4$.

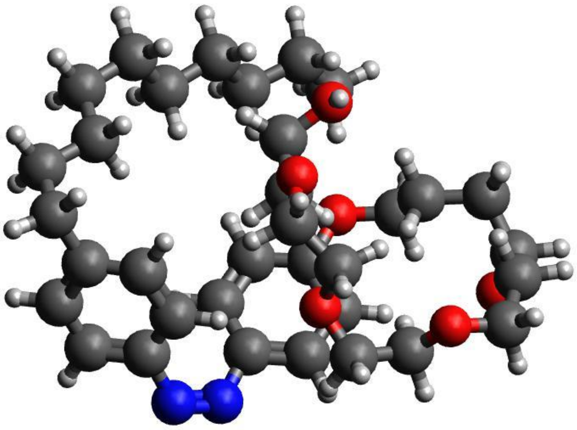

Figure S43. DFT optimized structure of the *Z*-isomer of $C_{10}AzoC_4E_4$.

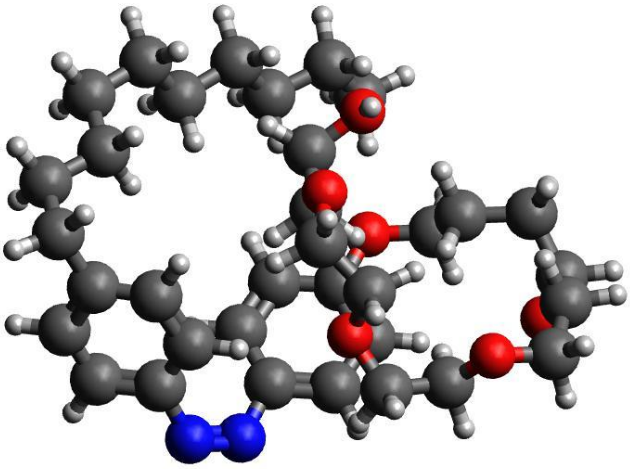

Figure S44. DFT-optimized structure of the *Z*-isomer of $C_{10}AzoC_4E_4$.

Table S14. Predicted isomerization enthalpies for AzoPS in the gas phase.

AzoPS	Predicted isomerization enthalpy / kJ mol^{-1}
$C_6AzoC_4E_4$	42.5
$C_8AzoC_4E_4$	36.8
$C_{10}AzoC_4E_4$	33.0

14 References

1. Houston, J.E., Kelly, E.A., Kruteva, M., Chrissopoulou, K., Cowieson, N., and Evans, R.C. (2019). Multimodal control of liquid crystalline mesophases from surfactants with photoswitchable tails. J Mater Chem C Mater *7*, 10945–10952. https://doi.org/10.1039/c9tc04079j.

2. Förster, S., Timmann, A., Konrad, M., Schellbach, C., Meyer, A., Funari, S.S., Mulvaney, P., and Knott, R. (2005). Scattering curves of ordered mesoscopic materials. Journal of Physical Chemistry B *109*, 1347–1360. https://doi.org/10.1021/jp0467494.

3. Israelachvili, J.N., Mitchell, D.J., and Ninham, B.W. (1976). Theory of self-assembly of hydrocarbon amphiphiles into micelles and bilayers. Journal of the Chemical Society, Faraday Transactions 2: Molecular and Chemical Physics *72*, 1525–1568. https://doi.org/10.1039/F29767201525.

4. Cowieson, N.P., Edwards-Gayle, C.J.C., Inoue, K., Khunti, N.S., Doutch, J., Williams, E., Daniels, S., Preece, G., Krumpa, N.A., Sutter, J.P., et al. (2020). Beamline B21: High-throughput small-angle X-ray scattering at Diamond Light Source. J Synchrotron Radiat *27*, 1438–1446. https://doi.org/10.1107/S1600577520009960.

5. Durchschlag, H., and Zipper, P. (1994). Calculation of the partial volume of organic compounds and polymers. In Ultracentrifugation, M. D. Lechner, ed., pp. 20–39.

6. Bannwarth, C., Ehlert, S., and Grimme, S. (2019). GFN2-xTB - An Accurate and Broadly Parametrized Self-Consistent Tight-Binding Quantum Chemical Method with Multipole Electrostatics and Density-Dependent Dispersion Contributions. J Chem Theory Comput *15*, 1652–1671. https://doi.org/10.1021/acs.jctc.8b01176.

7. Tanford, C. (1974). Thermodynamics of Micelle Formation: Prediction of Micelle Size and Size Distribution. Proc Natl Acad Sci U S A *71*, 1811–1815.

8. Pracht, P., Bohle, F., and Grimme, S. (2020). Automated exploration of the low-energy chemical space with fast quantum chemical methods. Physical Chemistry Chemical Physics *22*, 7169–7192. https://doi.org/10.1039/c9cp06869d.

9. Takeshita, K., Hirota, N., and Terazima, M. (2000). Enthalpy changes and reaction volumes of photoisomerization reactions in solution: azobenzene and p-coumaric acid. J Photochem Photobiol A Chem *134*, 103–109.

10. Brandenburg, J.G., Bannwarth, C., Hansen, A., and Grimme, S. (2018). B97-3c: A revised low-cost variant of the B97-D density functional method. Journal of Chemical Physics *148*. https://doi.org/10.1063/1.5012601.

11. Merino, E., and Ribagorda, M. (2012). Control over molecular motion using the *cis* – *trans* photoisomerization of the azo group. Beilstein Journal of Organic Chemistry *8*, 1071–1090. https://doi.org/10.3762/bjoc.8.119.

12. Franzke, Y.J., Holzer, C., Andersen, J.H., Begušić, T., Bruder, F., Coriani, S., Della Sala, F., Fabiano, E., Fedotov, D.A., Fürst, S., et al. (2023). TURBOMOLE: Today and Tomorrow. Preprint at American Chemical Society, https://doi.org/10.1021/acs.jctc.3c00347 https://doi.org/10.1021/acs.jctc.3c00347.

13. Agapito, F., Costa Cabral, B.J., and Martinho Simões, J.A. (2005). Carbon-hydrogen bond dissociation enthalpies in ethers: A theoretical study. Journal of Molecular Structure: THEOCHEM *719*, 109–114. https://doi.org/10.1016/j.theochem.2005.01.028.

14. Rosen, M.J., Cohen, A.W., Dahanayake, M., and Hua, X.Y. (1982). Surface and thermodynamic properties of 2-dodecyloxypoly(ethenoxyethanol)s, C12H25(OC2H4)x OH, in aqueous solution. Journal of Physical Chemistry *86*, 541–545. https://doi.org/10.1021/j100393a025.

15. Houston, J.E., Kelly, E.A., Kruteva, M., Chrissopoulou, K., Cowieson, N., and Evans, R.C. (2019). Multimodal control of liquid crystalline mesophases from surfactants with photoswitchable tails. J Mater Chem C Mater *7*, 10945–10952. https://doi.org/10.1039/c9tc04079j.
